# Seroprevalence, cross antigenicity and circulation sphere of bat-borne hantaviruses revealed by serological and antigenic analyses

**DOI:** 10.1371/journal.ppat.1007545

**Published:** 2019-01-22

**Authors:** Lin Xu, Jianmin Wu, Qi Li, Yamei Wei, Zhizhou Tan, Jianqiu Cai, Huancheng Guo, Ling’en Yang, Xiaohong Huang, Jing Chen, Fuqiang Zhang, Biao He, Changchun Tu

**Affiliations:** 1 Key Laboratory of Jilin Province for Zoonosis Prevention and Control, Institute of Military Veterinary Medicine, Academy of Military Medical Sciences, Changchun, Jilin, China; 2 Guangxi Key Laboratory of Veterinary Biotechnology, Guangxi Veterinary Research Institute, Nanning, Guangxi, China; 3 Institute for Viral Disease Prevention and Control, Hebei Province Center for Disease Prevention and Control, Shijiazhuang, Hebei, China; 4 College of Animal Science, Fujian Agriculture and Forestry University, Fuzhou, Fujian, China; 5 Institute of Animal Health, Guangdong Academy of Agricultural Science, Guangzhou, Guangdong, China; 6 Center for Disease Control and Prevention of Southern Theater Command, Kunming, Yunnan, China; 7 Jiangsu Co-innovation Center for Prevention and Control of Important Animal Infectious Diseases and Zoonosis, Yangzhou University, Yangzhou, Jiangsu, China; Johns Hopkins Bloomberg School of Public Health, UNITED STATES

## Abstract

Bats are newly identified reservoirs of hantaviruses (HVs) among which very divergent HVs have been discovered in recent years. However, their significance for public health remains unclear since their seroprevalence as well as antigenic relationship with human-infecting HVs have not been investigated. In the present study archived tissues of 1,419 bats of 22 species from 6 families collected in 5 south and southwest provinces in China were screened by pan-HV RT-PCR following viral metagenomic analysis. As a result nine HVs have been identified in two bat species in two provinces and phylogenetically classified into two species, Laibin virus (LAIV, ICTV approved species, 1 strain) and Xuan son virus (XSV, proposed species, 8 strains). Additionally, 709 serum samples of these bats were also analyzed by ELISA to investigate the seroprevalence and cross-reactivity between different HVs using expressed recombinant nucleocapsid proteins (rNPs) of LAIV, XSV and Seoul virus (SEOV). The cross-reactivity of some bat sera were further confirmed by western blot (WB) using three rNPs followed by fluorescent antibody virus neutralization test (FAVNT) against live SEOV. Results showed that the total HV seropositive rate of bat sera was 18.5% (131/709) with many cross reacting with two or all three rNPs and several able to neutralize SEOV. WB analysis using the three rNPs and their specific hyperimmune sera demonstrated cross-reactivity between XSV/SEOV and LAIV/XSV, but not LAIV/SEOV, indicating that XSV is antigenically closer to human-infecting HVs. In addition a study of the distribution of the viruses identified an area covering the region between Chinese Guangxi and North Vietnam, in which XSV and LAIV circulate within different bat colonies with a high seroprevalence. A circulation sphere of bat-borne HVs has therefore been proposed.

## Author summary

Some HVs are life-threatening pathogens predominantly carried and transmitted by rodents. In recent years bat-borne HVs have been identified in a broad range of bat species. To understand their significance to public health the present study conducted extensive investigations on genetic diversity, seroprevalence, distribution and cross antigenicity of bat-borne HVs in south and southwest China. The results provide the first profiling of cross-reactivity between bat-borne and human-infecting HVs, demonstrating that some bat sera can neutralize SEOV in cell culture. They also revealed that divergent bat-borne HVs co-exist and are widely distributed in Chinese Guangxi/Yunnan as well as in north Vietnam, resulting in identification of an area between China and Vietnam in which natural circulation of bat-borne HVs is maintained. Given the existence of bat-borne HVs genetically and antigenically close to human-infecting HVs, the need for extensive future studies is emphasized in order to assess the potential risk of these viruses to public health.

## Introduction

Hantaviruses (HVs), members of the genus *Orthohantavirus* within the family *Hantaviridae* in the order *Bunyavirales*, are responsible for two major life-threatening diseases in humans: hemorrhagic fever with renal syndrome (HFRS) in Eurasia and hantavirus cardiopulmonary syndrome (HCPS) in the Americas [1]. Every year around 100,000 HFRS cases and 1,000 HCPS cases are reported worldwide [2]. China suffers severely from epidemic HFRS; in 2017 alone, official statistics reported 11,262 cases with 64 deaths [3].

HVs are predominantly carried and transmitted by rodents, but insectivores and bats have also been reported as hosts. Several bat-borne HVs are presently known, which show large genetic diversities from currently known rodent- and insectivore-borne HVs. The first reported bat-borne HVs, Magboi virus (MGBV) and Mouyassué virus (MOUV), were identified respectively in Sierra Leone and Côte d’Ivoire of Africa in 2012 [4, 5]. Then two bat-borne HVs, Longquan virus (LQUV) and Huangpi virus (HUPV), were reported in China in 2013 [6], followed by the detection of Xuan son virus (XSV) at three locations in North Vietnam [7, 8]. We reported the first complete genome of a bat-borne HV, Laibin virus (LAIV), identified from a black-bearded tomb bat in Guangxi Province of China in 2015 [9, 10]. Since then three more complete genomes of bat-borne HVs, Makokou virus (MAKV), Quezon virus (QZNV) and Brno virus (BRNV) have been reported sequentially in 2016, in Central Africa (Gabon), Southeast Asia (Philippines) and Central Europe (Czech Republic), respectively [11–13]. Most recently, a sister lineage of MOUV was detected in dried blood samples from bats in Eastern Africa (Ethiopia) in 2017 [14]. Of these HVs only three, Laibin, Longquan and Quezon viruses were approved as bat-borne HV species within genus *Orthohantavirus* in the 10^th^ report of International Committee on Taxonomy of Viruses (ICTV) released in 2017 [15]. Phylogenetic analysis of bat-borne HVs has indicated that bats might be the natural original hosts of HV: i.e., the viruses first appeared in bats or insectivores, then emerged in rodents [6, 16–19]. However, due to lack of sufficient bat-borne HV genomic sequences, their evolutionary phylogeny and genetic diversity as well as biological features are poorly understood.

HVs are enveloped and spherical in shape although pleomorphic forms are also found with the diameters ranging from 80–120 nm. Within the capsid is a tripartite negative-stranded RNA genome consisting of small (S), medium (M) and large (L) segments with a total length of about 11.8 kb, respectively encoding nucleocapsid protein (NP), glycoprotein (GP, a precursor for two viral surface glycoproteins, Gn and Gc) and RNA-dependent RNA polymerase (RdRp) [1]. The NP is multifunctional and plays an essential role in viral replication, not only binding viral RNA strands to form a ribonucleoprotein (RNP) to prevent RNA from degradation, but also regulating virus replication and assembly [1, 20, 21]. NP is also the main target for the earliest immune response. Its coding gene is much more conserved than the GP gene, and is therefore commonly used as a diagnostic antigen for HV detection [22–25]. Different serotypes of HVs can be determined by an at least four-fold difference in two-way cross neutralization tests, and it has been reported that serotype-specific as well as group-common and genus-common epitopes can be found in the NP. Cross-reactivity has been found between different serotypes of HVs in rodents and insectivores [26–28]. However, the serological and antigenic relationships between bat- and rodent- or insectivore-borne HVs have not yet been studied.

South and southwest China have a high density of bat population consisting of a large number of diverse species. Recently, investigations on bat viruses in this area have revealed many novel viruses, such as coronaviruses [29–34], filoviruses [35, 36] and group A rotaviruses (RVA) [37, 38]. Among these, some bat-borne coronaviruses [29, 30, 33] and RVAs [38] have been found to cross species, causing outbreaks of emerging infectious diseases in human and pigs. South and southwest China are also the major epidemic areas of HFRS with all transmission events associated with exposure to rodents [39]. Although increasing number of HVs have been identified in bats, no investigation has been shown their seroprevalence and antigenic characters. The implication of bat-borne HVs to public health is still unclear. In present study, we have conducted systematic etiological and serological investigation on bat-borne HVs in south and southwest China, revealing the antigenic relationships between bat-borne and human-infecting HVs and identifying a geographic region between southwest China and north Vietnam in which divergent bat-borne HVs circulate.

## Results

### Hantaviral RNA detection and attempts for virus isolation

Following viral metagenomic analyses of bat intestines and lungs, 18 contigs with lengths of 110–726 nucleotides (nt) from Laibin (LB), Baise (BS) and Pu’er (PE) cities (see S1 Fig) were annotated to HVs. The highest nt identities of these (72–99%) were shared with the M or L segments of LAIV or XSV (S1 Table). By pan-HV PCR screening of all intestines and lung tissues, only lung tissue of 9 bats were found to be positive. One of 39 (2.6%) black-bearded tomb bats (*Taphozous melanopogon*) in BS, Guangxi, 5 of 55 (9.1%) pomona roundleaf bats (*Hipposideros pomona*) in LB, Guangxi, and 3 of 40 (7.5%) pomona roundleaf bats in PE, Yunnan were also positive (Table 1). The viral sequence determined in black-bearded tomb bats in BS showed a 97% nt identity with previously reported LAIV BT20 [9], and were therefore considered an LAIV variant, BT33. The rest eight from LB and PE showed 93% and 82% nt identities with XSVs identified from Vietnam [7, 8], indicating they were all XSV variants and therefore respectively named XSV AR18, AR19, AR23, AR28, AR30, PR10, PR15 and PR30.

**Table 1:** Positive rate of HV of bat sera and tissue in different provinces.

Bat species	Positive rate % (Positive /Total)^a^				
	Yunnan	Guangxi	Guangdong	Fujian	Zhejiang
*Rh*^b^. *affinis*	**30.0 (6/20)**	**22.5 (9/40)**		**20.6 (14/68)**	
	0 (0/20)	0 (0/65)		0 (0/85)	
*Rh*. *hipposideros*					
		0 (0/15)			
*Rh*. *macrotis*					
		0 (0/2)			
*Rh*. *pearsonii*		0 (0/2)			
		0 (0/37)			
*Rh*. *pusillus*					
	0 (0/1)	0 (0/9)	0 (0/5)		
*Rh*. *sinicus*				**5.9 (2/34)**	
				0 (0/50)	
*Rh*. *thomasi*		**28.6 (2/7)**			
		0 (0/22)			
*Rh*. *ferrumequinum*					**10.6 (5/47)**
					0 (0/47)
*Hi*. *armiger*		**3.1 (1/32)**		**10.0 (1/10)**	
		0 (0/43)	0 (0/11)	0 (0/10)	
*Hi*. *cineraceus*					
		0 (0/50)	0 (0/9)		
*Hi*. *larvatus*		**6.3 (5/79)**			
		0 (0/186)	0 (0/68)		
*Hi*. *pomona*		**40.0 (2/5)**			
	**7.5 (3/40)**	**3.0 (5/168)**			
*Hi*. *pratti*					
		0 (0/1)			
*Hi*. *turpis*		0 (0/8)			
		0 (0/8)			
*As*. *stoliczkanus*		**50.0 (1/2)**			
		0 (0/4)			
*Mi*. *australis*		0 (0/15)			
		0 (0/52)			
*Mi*. *schreibersii*		**20.0 (7/35)**			
		0 (0/77)			
*My*. *horsfieldii*				**21.7 (5/23)**	
				0 (0/37)	
*Sc*. *kuhlii*		**10.2 (11/108)**			
		0 (0/135)			
*Ta*. *melanopogon*		**28.1 (9/32)**			
		**1.4 (1/74)**			
*Ro*. *leschenaulti*	**35.9 (51/142)**				
			0 (0/37)		
*Cy*. *sphinx*					
			0 (0/51)		
Total	**35.2 (57/162)**	**12.9 (47/365)**		**16.3 (22/135)**	**10.6 (5/47)**
	**4.9 (3/61)**	**0.6 (6/948)**	0 (0/181)	0 (0/182)	0 (0/47)

^a^ For each bat species the upper (shaded) and lower rows provide serum and tissue information respectively. Positive results are identified in bold. Seropositive rates were based on ELISA results.

^b^ Abbreviations of bat genera: *Hi*., *Hipposideros*; *As*., *Aselliscus*; *Rh*., *Rhinolophus*; *Sc*., *Scotophilus*; *Mi*., *Miniopterus*; *My*., *Myotis*; *Ta*., *Taphozous*; *Ro*., *Rousettus*; *Cy*., *Cynopterus*.

To isolate infectious viruses, homogenized lung tissues of five XSV ARs and three XSV PRs were separately pooled. The two pooled samples, along with one LAIV BT33 positive lung tissue were thoroughly homogenized by grinding and their filtered supernatants were incubated with African green monkey kidney (Vero) and the E6 clone and baby hamster kidney (BHK-21) cell cultures. During five passages, no CPE was observed, and RT-PCR analyses of all passaged cultures were negative, with no HV isolated.

### Whole genome sequencing and comparison

To gain genetic insight into the HVs, the full genomes of LAIV BT33, XSV AR18, AR23 and PR15 were sequenced and analyzed using previously reported methods [9, 40]. As shown in Table 2, three gene segments of LAIV BT33 had the same sizes as previously reported LAIV BT20 [9]. Three segments of XSV AR18 and AR23 had exactly the same size (1,753 nt of S, 3,751 nt of M and 6,521 nt of L), while PR15 had similar sized S (1,743 nt) and L (6,522 nt), but its M segment was shorter (3,584 nt) than those of XSV AR18 and AR23, resulting from a 50-aa deletion at the 5’ terminal of the coding sequence, corresponding to 6–55 aa of Gn protein. This deletion was confirmed by repeated RT-PCR and sequencing. Currently the function of Gn is largely unknown and 1–17 aa is the signal peptide of Gn responsible for translocation of Gn to Golgi [41, 42], therefore the deletion may have impact on Gn location. In addition, the highly conserved motif WAASA (polyprotein-recognized pentapeptide) in the M segment of HV was observed in all four strains, but the ORF in the S segment of some HVs (such as Puumala, Tula and Andes viruses) encoding a 7–12 KDa nonstructural protein (NSs) which functioned as an interferon antagonist were not found [43–45]. Sequence comparison of the four strains with other bat-borne HVs available in GenBank (Table 2) showed that LAIV BT33 shared the highest (98.4–98.6% nt and 99.2–100.0% aa) identities with LAIV BT20 in its three genomic (full-length) segments and low identities with other bat-borne HVs, (49.6–75.4% nt and 45.8–87.3% aa identities in full or partial gene segments), indicating that it is a variant of LAIV. XSV AR18 and AR23 shared the highest (91.8–93.4% nt and 99.0–100.0% aa) identities with the XSV strain F42682 (partial gene segments, full-length not available) and XSV PR15 the highest (82.8–84.9% nt and 97.9–99.1% aa) identities with XSV F44601 (partial gene segments, full-length not available), indicating that they are novel variants of XSV. Full-length genomic sequence comparison of the four strains with those of rodent-and insectivore-borne HVs showed that bat-borne HVs of the present study had very low nt (43.3–66.6%) and aa (40.0–67.6%) similarities to rodent-and insectivore-borne HVs (S3 Table).

**Table 2:** The 9 bat-borne HVs identified to date and their sequence comparison with new viruses obtained in the present study.

Virus	Strain	Country	Bat species	Segment	Accession NO.	Length (nt/aa) ^a^	Identity (nt/aa)		
							BT33	AR18 ^b^	PR15
LAIV	BT20	China	*Ta*. ^c^ *melanopogon*	S	KM102247	**1,935/427**	98.5/100.0	59.9/78.4	58.5/78.4
				M	KM102248	**3,608/1,127**	98.4/99.2	64.3/71.9	61.7/72.3
				L	KM102249	**6,531/2,145**	98.6/99.7	72.5/80.7	72.6/80.8
	BT33	China	*Ta*. *melanopogon*	S	KY662264	**1,935/427**	-	59.8/78.4	58.6/78.4
				M	KY662265	**3,608/1,127**	-	64.3/72.2	61.7/72.5
				L	KY662266	**6,531/2,145**	-	72.6/80.7	72.7/80.8
XSV	AR18	China	*Hi*. *pomona*	S	KY662267	**1,753/427**	59.8/78.4	-	79.7/96.7
				M	KY662268	**3,751/1,128**	64.3/72.2	-	76.1/93.6
				L	KY662269	**6,521/2,145**	72.6/80.7	-	79.7/94.3
	PR15	China	*Hi*. *pomona*	S	KY662273	**1,743/427**	58.6/78.4	79.7/96.7	-
				M	KY662274	**3,584/1,078**	61.7/72.5	76.1/93.6	-
				L	KY662275	**6,522/2,145**	72.7/80.8	79.7/94.3	-
	F42682	Vietnam	*Hi*. *pomona*	S	KF704709	**1,752/427**	59.7/78.3	92.5/100.0	79.9/96.3
				M	KJ000538	663/221	71.6/74.7	93.4/100.0	79.6/95.9
				L	KF704714	1,160/386	75.0/85.8	91.8/99.0	80.1/96.9
	F44601	Vietnam	*Hi*. *pomona*	S	KF704712	**1,728/427**	58.4/78.1	80.3/97.2	84.9/99.1
				M	KJ000539	663/221	70.6/74.2	80.5/96.8	83.3/98.6
				L	KF704717	1,160/386	75.4/87.3	81.0/95.3	82.8/97.9
	VN1982B4	Vietnam	*Hi*. *pomona*	S	KC688335	499/166	68.7/74.1	88.0/99.4	79.0/94.6
				M	KU976427	3,388/1,009	65.4/71.3	83.8/94.0	76.7/91.3
				L	JX912953	4,582/1,527	71.9/79.4	85.2/97.2	78.6/94.2
QZNV	MT1720/1657	Philippines	*Ro*. *amplexicaudatus*	S	KU950713	**1,830/429**	57.3/63.1	57.3/61.7	57.7/61.7
				M	KU950714	**3,772/1,133**	56.9/55.4	59.9/54.9	58.9/56.0
				L	KU950715	**6,556/2,147**	66.4/69.0	67.0/70.4	67.1/70.2
BRNV	7/2012/CZE	Czech	*Ny*. *noctula*	S	KX845678	**1,269/423**	59.7/56.9	59.7/55.9	60.2/55.5
				M	KX845679	**3,408/1,136**	56.8/45.8	56.4/45.9	56.8/45.6
				L	KX845680	**6,432/2,144**	67.2/66.9	66.5/65.0	65.8/65.3
MAKV	GB303	Gabon	*Hi*. *ruber*	L	KT316176	3,580/1,173	74.6/85.3	74.1/83.8	74.0/84.0
LQUV	Ra-10	China	*Rh*. *affinis*	S	JX465413	**1,565/423**	50.1/61.0	58.4/59.6	58.0/58.9
				M	JX465396	**3,618/1,133**	51.3/47.2	56.3/46.7	58.7/46.3
				L	JX465379	324/107	70.7/71.3	70.4/71.3	71.0/69.4
	Rs-32	China	*Rh*. *sinicus*	S	JX465422	**1,564/423**	49.6/61.7	58.0/60.6	57.8/59.6
				M	JX465402	**3,619/1,133**	51.2/47.2	56.2/46.7	58.7/46.3
				L	JX465388	324/107	70.7/71.3	70.4/71.3	71.0/69.4
	Rm-180	China	*Rh*. *monoceros*	S	JX465419	**1,564/423**	50.0/61.7	58.4/60.6	58.1/59.6
				L	JX465385	324/107	70.4/71.3	69.8/71.3	70.4/69.4
HUPV	Pa-1	China	*Pi*. *abramus*	S	JX473273	1,115/271	55.2/62.5	56.5/61.4	56.3/60.3
				L	JX465369	343/114	69.4/79.8	66.8/71.1	67.1/72.8
MOUV	2455	Ethiopia	*Ne*. *capensis*	L	KX184829	237/79	67.1/72.2	66.2/68.4	68.8/70.9
	KB576	Cote d'Ivoire	*Ne*. *nanus*	L	JQ287716	1,691/563	70.3/77.6	70.3/77.8	71.1/78.0
MGBV	1209	Sierra Leone	*Nyc*. *hispida*	L	JN037851	414/137	71.5/76.6	71.5/75.2	73.0/72.3

^a^ Full-length sequences are given in bold, although that of BRNV does not include non-translating regions.

^b^ AR18 and AR23 shared 99.2–100% nt and aa identities, and therefore the latter is not included in the table.

^c^ Abbreviation of bat genera: *Ta*., *Taphozous*; *Hi*., *Hipposideros*; *Ro*., *Rousettus*; *Ny*., *Nyctalus*; *Rh*., *Rhinolophus*; *Pi*., *Pipistrellus*; *Ne*., *Neoromicia*; *Nyc*., *Nycteris*.

### Phylogenetic analyses

**Fig 1:**
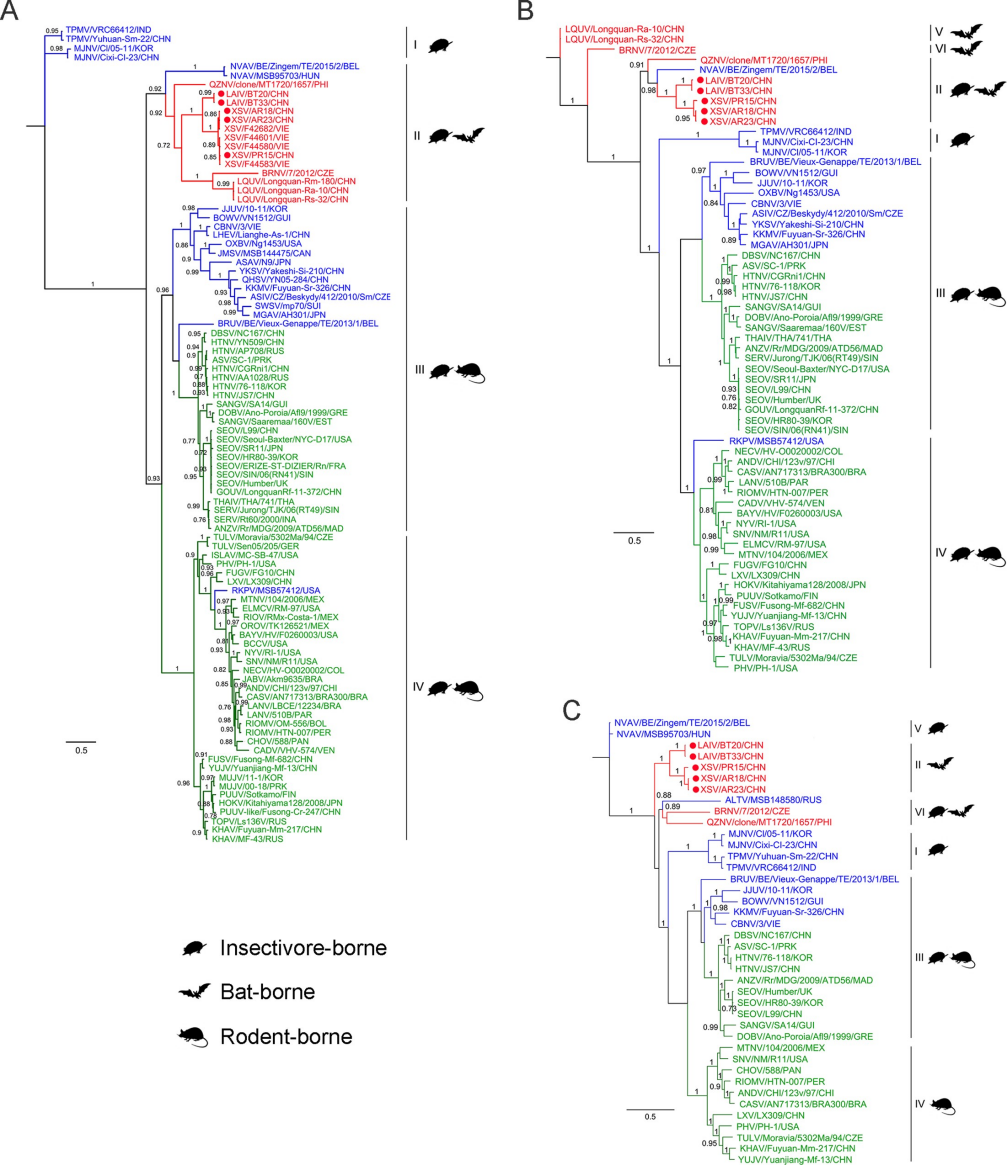
**Phylogenetic trees generated based on the encoding sequences of NP gene segment (~427aa, A), GP (~1127 aa, B) and RdRp (~2145 aa, C)**. Bootstrap values of 1,000 replicates (>0.7) are shown and the scale bars indicate nucleotide substitutions per site. Sequences of bat-borne HVs are shown in red with the four strains of the present study marked by filled red circles. Sequences of insectivore- and rodent-borne HVs are shown in blue and green respectively. Definitions of virus abbreviations and their GenBank accession numbers are in S4 Table.

To construct phylogenetic relationships, 92, 63 and 40 complete coding sequences of hantaviral NP, GP and RdRp respectively were used. Currently there are only 14 NP, 9 GP and 7 RdRp complete sequences of bat-borne HVs available in GenBank. As shown in Fig 1, rodent- and insectivore-borne HVs, except for Nova virus (NVAV) [18, 46] and Altai virus (ALTV), showed a similar topology, which classified them within clades I, III and IV in all three trees. Bat-borne HVs showed different topology structures, however, which were all clustered together within clade II in the NP tree, but within three clades (II, V and VI) in the GP tree, or within two clades (II and VI) in the RdRp tree. It is interesting to note that two insectivore-borne HVs, NVAV and ALTV, respectively identified in *Talpa* moles and *Sorex* shrews, were genetically closer to bat-borne than to insectivore-borne HVs in the NP and GP trees [18, 46].

### Cross-reactivity of LAIV, XSV and SEOV from NP-based serology

**Fig 2:**
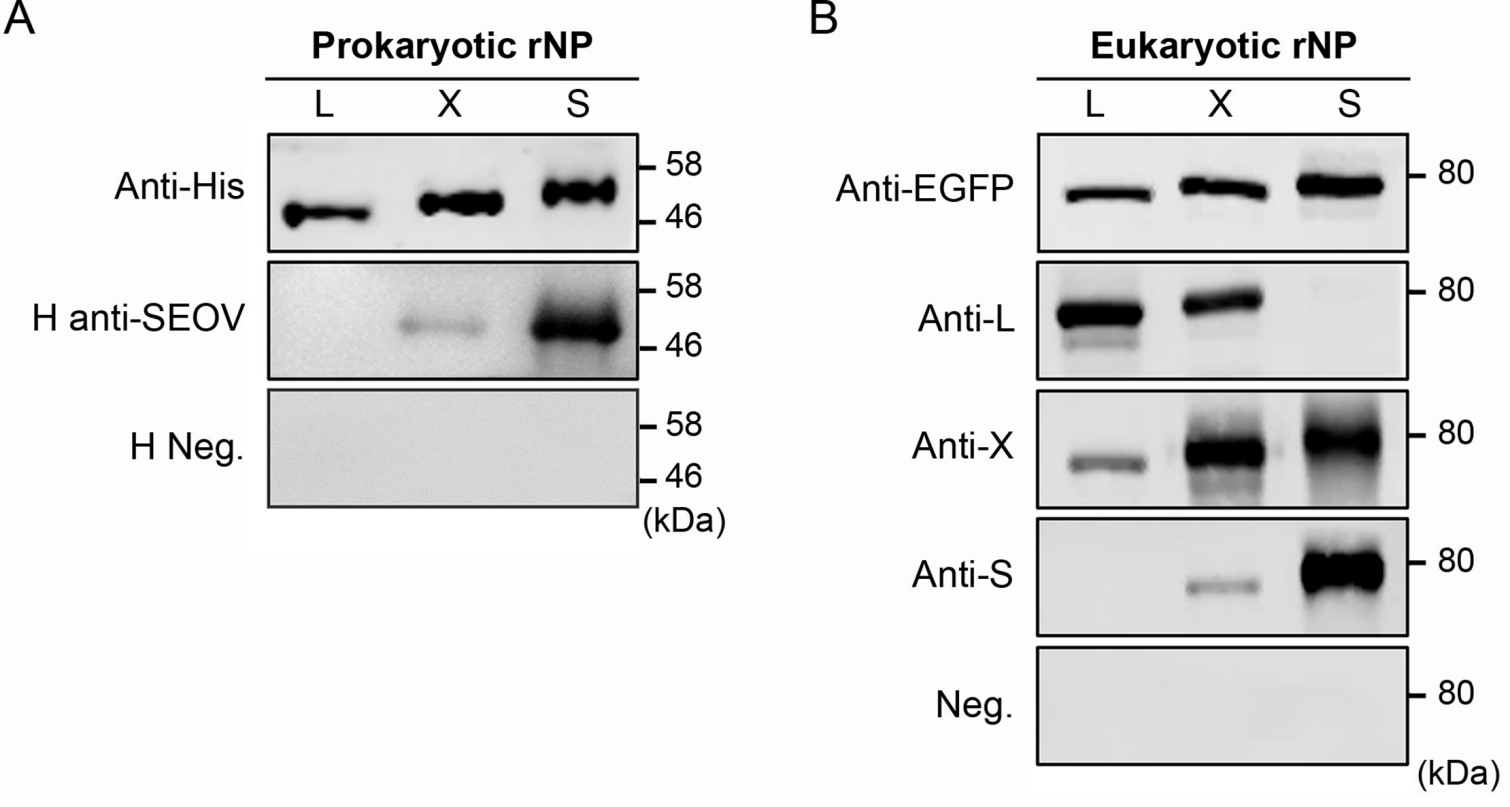
rNP cross-reactivity among LAIV, XSV and SEOV. Western blot was used to characterize the reactivity of (A) prokaryotically expressed rNPs by SEOV-convalescent human serum (H anti-SEOV) and negative human serum (H Neg.), and (B) eukaryotically expressed rNPs by three specific anti-rNP hyperimmune sera against LAIV (Anti-L), XSV (Anti-X) and SEOV (Anti-S) with normal mouse serum (Neg.) as a negative antiserum control. Anti-6X His-tag monoclonal antibody (Anti-His) and anti-EGFP monoclonal antibody (Anti-EGFP) were used to normalize the rNP loading in each lane (0.2 μg/lane for A and 8 μg/lane in B). Abbreviations: L, LAIV-rNP; X, XSV-rNP; S, SEOV-rNP.

To characterize the antigenic relationship between bat- and human-infecting HVs the entire NPs of LAIV BT33, XSVAR18 and SEOV GuangzhouRn36 were expressed in *E*. *coli* and purified. Polyclonal anti-serum against the rNPs (named anti-L, anti-X and anti-S, respectively, for LAIV, XSV and SEOV) were prepared by immunization of mice, resulting in titers by ELISA of 8,000×, 4,000× and 4,000× respectively. Western blot (WB) analyses showed that SEOV-convalescent human serum (H anti-SEOV) had a significant cross-reactivity with the rNP of XSV but not LAIV, as well as the rNP of SEOV (Fig 2A). To further characterize this cross antigenicity, three NPs were eukaryotically expressed with an EGFP tag. Further WB analyses with the three anti-rNP sera showed that anti-L reacted strongly with the eukaryotic rNP of XSV but not with that of SEOV, in addition to a very strong reactivity with its own LAIV rNP (Fig 2B). In contrast, anti-S showed only a weak cross-reactivity with eukaryotic rNP of XSV and not at all with that of LAIV, although with very strong reactivity with its own SEOV NP. It is interesting to note that anti-X had cross-reactivity with eukaryotic rNPs of both LAIV and SEOV (weak for LAIV and strong for SEOV), in addition to a very strong reactivity with its own XSV rNP. This result unexpectedly showed that bat-borne HVs do share cross reactivity with human-infecting HVs and that significant differences of antigenicity do exist in different bat-borne HVs. In our study XSV was antigenically closer to SEOV than LAIV.

### Seroprevalence of HVs in bat sera

**Fig 3:**
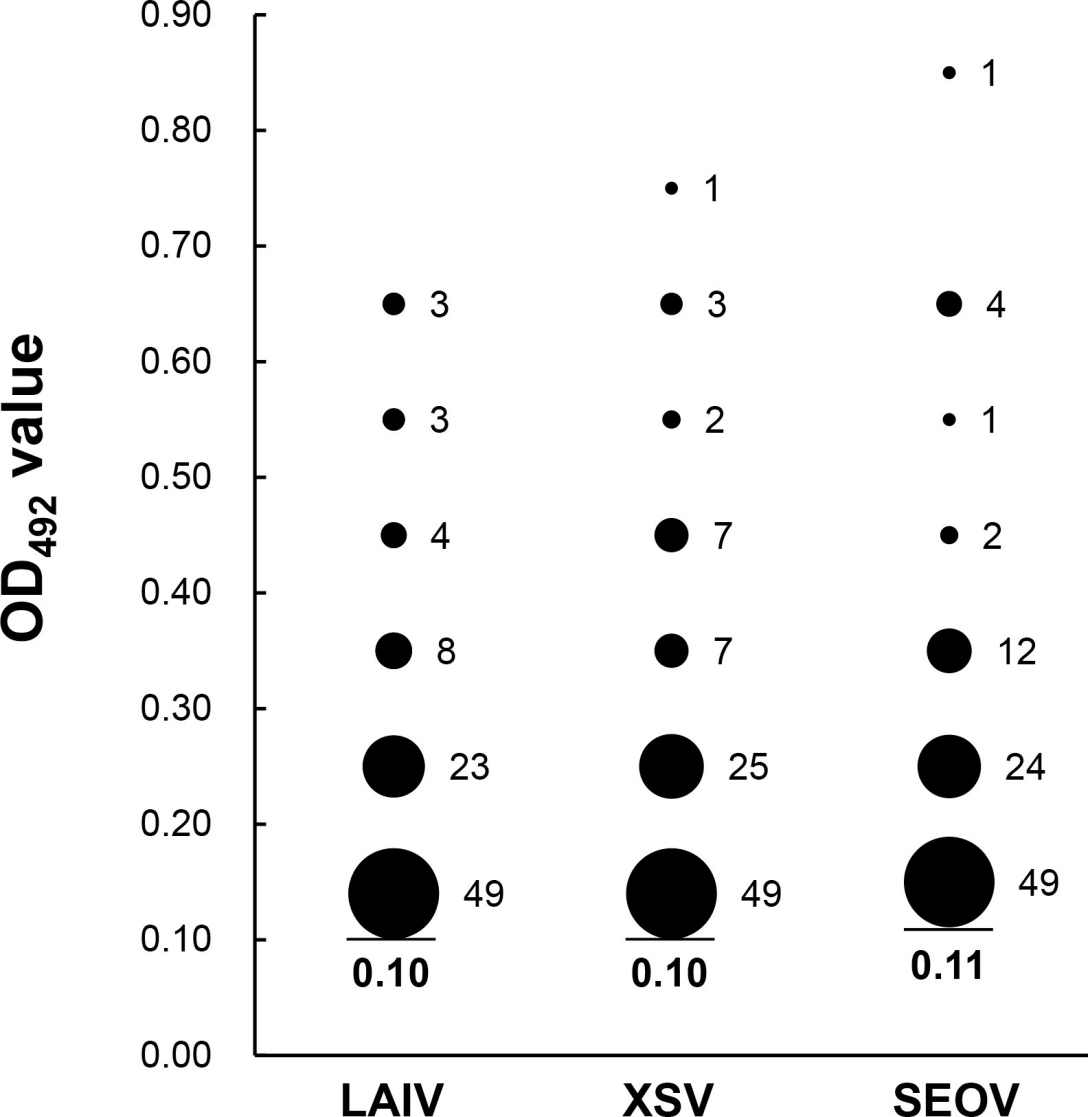
Distribution of OD_492_ values of positive bat sera (Y axis) against the rNP of each virus (X axis).

**Fig 4:**
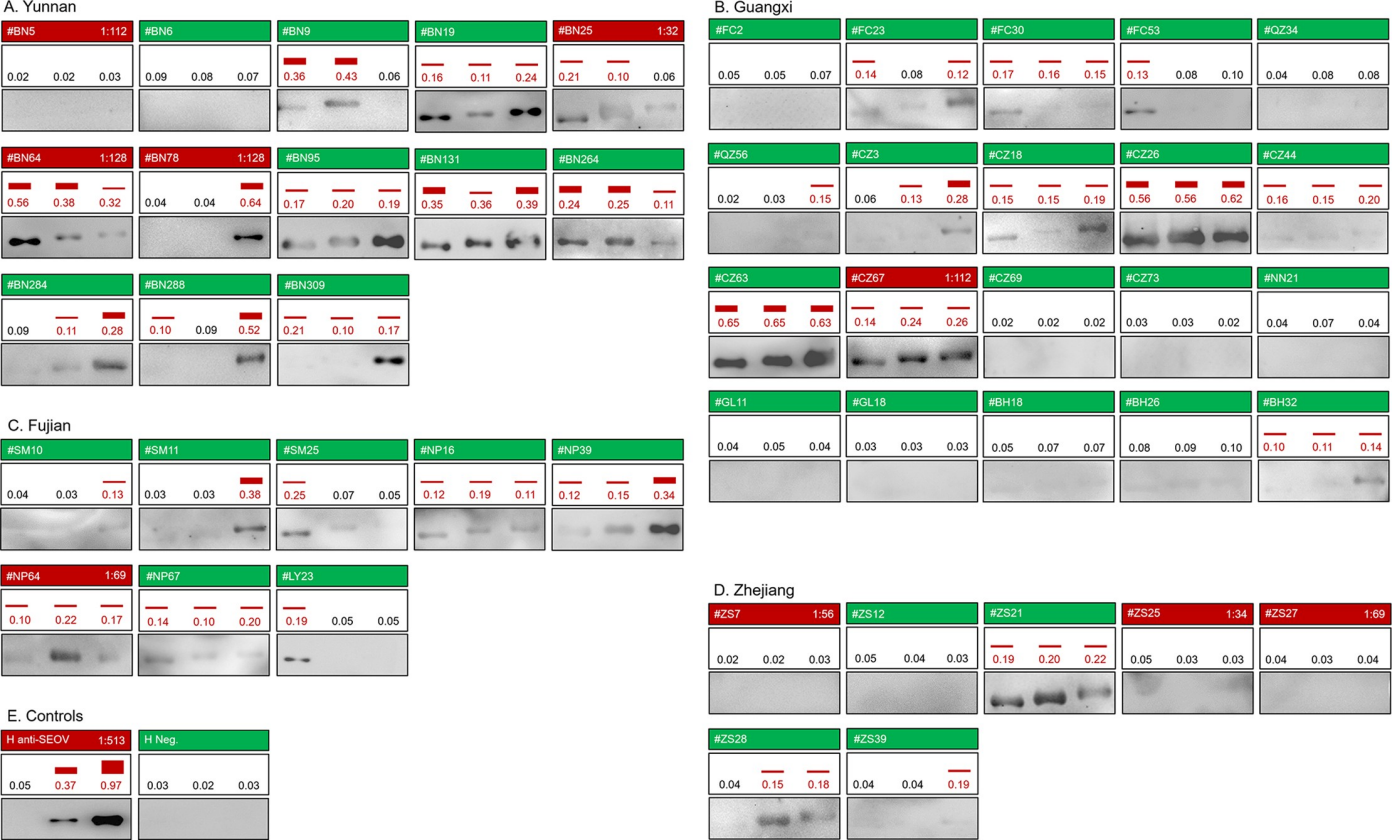
Serological results of 48 bat sera and 2 control human sera. Bat sera in four provinces: (A) Yunnan (n = 13), (B) Guangxi (n = 20), (C) Fujian (n = 8) and (D) Zhejiang (n = 7). (E) two human sera as positive (H anti-SEOV) and negative (H Neg.) serum controls. Each square represents one serum, consisting of upper (FAVNT), middle (ELISA) and lower (WB) rectangular boxes indicating the assay method. Numbering of each sample is to the left of the FAVNT box. FAVNT positive sera are noted in red, with the titer on the right, while the negatives are in green (no titer). In the ELISA box, the OD_492_ value against the rNP of LAIV, XSV and SEOV are shown from left to right with the positive titers in red and negatives in black (no bar). In the WB box, 0.2 μg/lane rNP of LAIV, XSV and SEOV were separately loaded from left to right.

**Fig 5:**
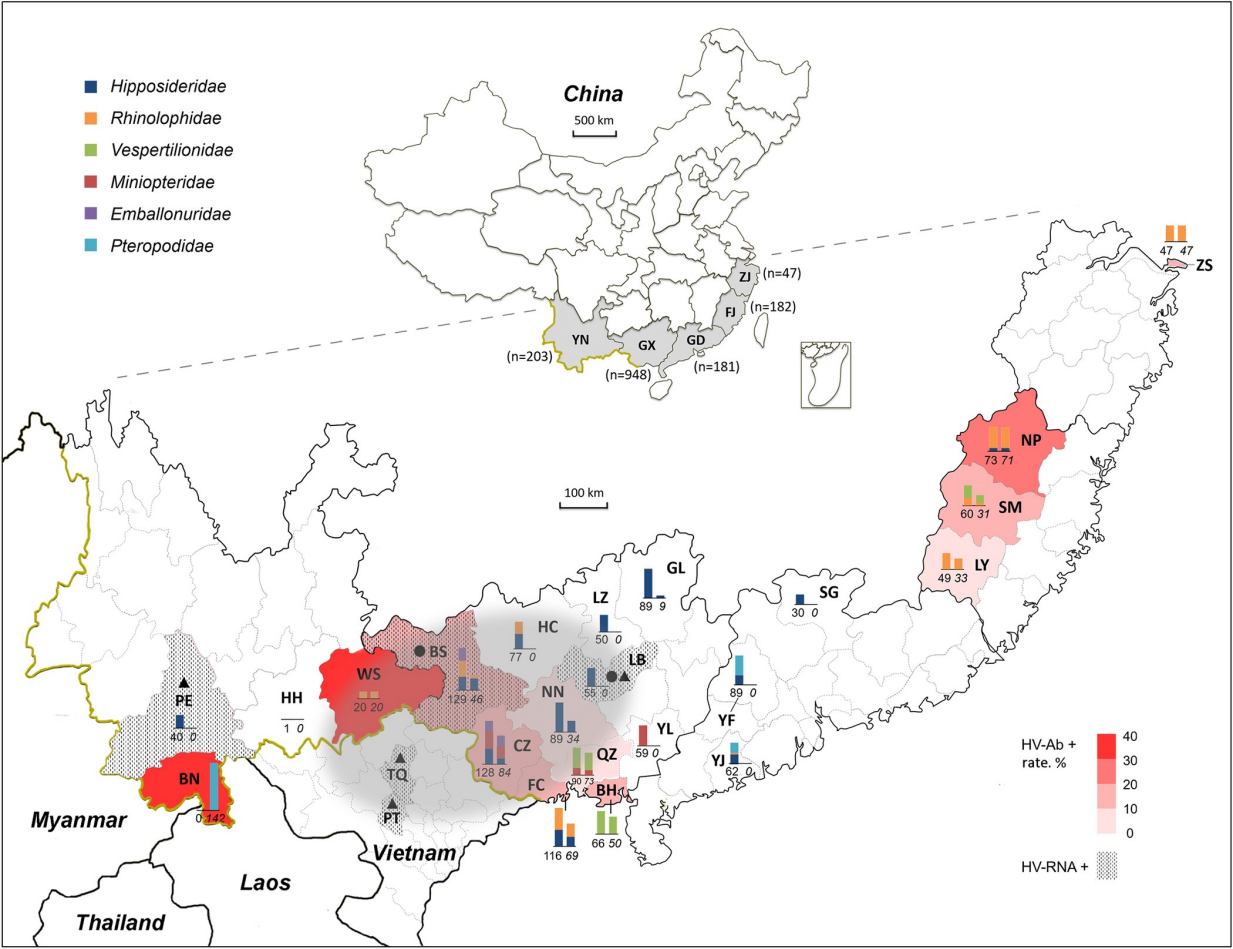
Information of sample collection and detection. Bat sera and/or tissues were collected from 22 cities in 4 provinces: Xishuangbanna (BN), Honghe (HH), Pu’er (PE) and Wenshan (WS) cities in Yunnan (YN) Province; Beihai (BH), Baise (BS), Chongzuo (CZ), Fangchenggang (FC), Guilin (GL), Hechi (HC), Laibin (LB), Liuzhou (LZ), Nanning (NN), Qinzhou (QZ) and Yulin (YL) cities in Guangxi (GX) Zhuang Autonomous Region; Shaoguan (SG), Yunfu (YF) and Yangjiang (YJ) cities in Guangdong (GD) Province; Nanping (NP), Longyan (LY) and Sanming (SM) cities in Fujian (FJ) Province; and Zhoushan (ZS) city in Zhejiang (ZJ) Province. The combined 2 bars represent numbers of bat samples collected in each city, either tissues (left) and serum (right). Total samples are listed under each bar. Lengths of color boxes in the bars represent the numbers of sampled bats at family level (color illustration is on upper left). The number of sampled bats in each province is shown on the upper Chinese map. Hantavirus seropositive rates (HV-Ab+ rate) in different cities are indicated by different shades of red. Bar scale of red color density on the lower right represents the rates from 0% to 40%. Filled circles and triangles indicated where LAIV and XSV were identified. The shaded grey oval region is the proposed main circulation sphere of bat-borne HVs.

Results of the serological assay of 709 bat sera by ELISA against the three viruses are shown in S2 Fig with 88 of them being further confirmed by WB (S3 Fig). Since no standard bat sera (either positive or negative) were available, the highest coincidence rate (CR) between WB and ELISA was used to determine OD_492_ ELISA positive cut-off values: 0.10, 0.10 and 0.11 at the highest CR value for each virus (87.5%, 86.4% and 86.4%, respectively, for LAIV, XSV and SEOV) (see S4 Fig). With such cut-offs, the κ test showed high levels consistence between the two methods with Z values being 7.0862 for LAIV, 6.8255 for XSV, and 6.9270 for SEOV (all p<0.0001), and the high κ values being 0.7260–0.7505. These results indicate that the established ELISA was valid to test the bat sera. Using these cut-offs, 131 of 709 (18.5%) bat sera were found to be HV antibody positive. Fig 3 shows the distribution of OD_492_ readings of the 131 positive bat sera, with most sera having OD_492_ readings between the cut-off and 0.30. To further determine antibody titers, the positive sera were 4-fold diluted from 100× to 1,600× and retested by ELISA. Results showed that most positive sera had titers of 100×, yet 18 sera reached 400×, with the H anti-SEOV at 1,600× (Fig 4). Of 131 positive sera, 55 (7.76%) showed cross-reactivity to all three viruses, 19 (2.7%) to both of LAIV and XSV, 9 (1.3%) to both XSV and SEOV, and 7 (1.0%) to both LAIV and SEOV, whereas sera reacted exclusively with one virus were only 9 (1.3%) with LAIV, 10 (1.4%) with XSV and 22 (3.1%) with SEOV. This further showed that seroprevalence of HVs in bats widely existed in four provinces (in Guangdong bat sera were not collected). As shown in Fig 5, among 13 cities with bat serum collection 12 were seropositive with levels from 5.5% to 35.9%. Of 16 bat species tested 13 had seropositive rates ranging from 4.8% to 50.0%.

### Cross neutralization determined by FAVNT

Of 88 bat sera tested by WB, 48 with sufficient volume were further tested for neutralizing antibody (NAb) titers against SEOV by the fluorescent antibody virus neutralization test (FAVNT). Results showed that nine bat sera (18.8%) from four provinces had NAb titers ranging from 32× to 128× (Fig 4), of which five were both WB and ELISA positive, with the other four negative for both. The positive control (H anti-SEOV) had an NAb titer of 513× (Fig 4). Of interest is that one serum from *Rousettus leschenaultii* bat in Xishuangbanna, BN78, had the highest NAb titer (128×) against SEOV and was WB and ELISA positive only for SEOV, not for LAIV or XSV. Results also showed that some WB and ELISA double-positive bat sera against the three viruses did not neutralize SEOV, including samples BN19, CZ63, CZ26, NP39 and ZJ61.

## Discussion

### Genetic diversity of bat-borne HVs

As bat-borne HVs have only recently been identified, there is insufficient sequence data at present to provide a comprehensive analysis of their genetic diversities. Apart from the four complete genomic sequences reported here (Table 2), the sequences of representative bat-borne HVs published to date in GenBank show that many are not of complete genomes or even of a full-length gene segment [5–7, 11]. Currently Laibin, Longquan and Quezon viruses are the only bat-borne HVs approved by ICTV so far [6, 9, 12]. Among completed genomes, there are the complete coding sequences of the three gene segments of BRNV from the Czech Republic [13]. Here we report the first genomic sequence of XSV and show that XSV and BRNV should be considered new HV species awaiting ICTV consideration. Fortunately, there are partial L gene sequences available for all bat-borne HVs, which allowed construction of a phylogenetic tree (using 314 bp), permitting comparison with rodent- and insectivore-borne HVs (S5 Fig). This showed that current bat-borne HVs can be classified into nine species as listed in Table 2, with almost every one having a specific bat genus as host. Of them XSV is the most notable bat-borne HV, which has been found in different locations in present and previous studies [7, 8], and showing significant nt variation although aa sequences of the genomic segments are conserved. The nt identity between currently identified XSV variants ranges from 76.1%-93.4% (Table 2). Furthermore the NP of XSV showed cross antigenicity with both SEOV and LAIV but the NP of LAIV showed no cross antigenicity with SEOV (Fig 2B), indicating that XSV is antigenically closer than LAIV to SEOV and therefore an ideal focus for gaining insight into the role of bat-borne HVs in public health. Meanwhile the closer relationship of insectivore-borne HVs NVAV and ALTV to bat-borne than to insectivore-borne HVs in the NP and GP trees (Fig 1) indicated that HVs from bats and insectivores could share the common ancestry for evolution [18, 46]. In general our sequence comparisons and phylogenetic analyses show that bat-borne HVs had broad genetic diversities and had evolved worldwide within an independent and diverse phylogroup. In this regard, more extensive studies obtaining more complete sequences in extended areas will undoubtedly identify more novel bat-borne HVs in future.

### Seroprevalence and cross antigenicity of bat-borne HVs

Although nine bat-borne HVs have been identified worldwide, the virus detection rate is low and in limited locations [5, 7, 9, 11]. The present study investigated bats in 22 cities, but the viral RNA was found in only three bat colonies in three cities, and RNA-positive rates were only 1.4% (1/74) for LAIV in BS, 3.0% (5/168) for XSV in LB and 7.5% (3/40) for XSV in PE (see Fig 5 and Table 1). In contrast, seropositive rates are higher: 28.1% (9/32) for LAIV and 40% (2/5) for XSV in BS and LB respectively (sera were not collected in PE) (Table 1). Low viral RNA detection rates have also been reported in previous publications with 5.6% (1/18) for MGBV in Sierra Leone [5], 3.1% (1/32) for LAIV BT20 in China [9], and 0.8% (1/123) for MAKV in Gabon [11]. Unfortunately, the seroprevalence was not reported in these publications. The antibody titers of bat sera against HVs in our study were rather low (the most were 100× and only 18 were 400×) as compared to those of rodent reservoirs which were usually higher and could reach 50,000 [24, 47], this might be ascribed to the higher diversity of V_H_ (especially FR3) in immunoglobulin genes of bats in comparison to those of mouse, swine and human [48].

The correlation between RT-PCR positivity and antibody positivity about hantavirus infection in rodents or shrews were reported [49, 50]. Song *et al*. reported that a certain proportion, although not all, of Ussuri white-toothed shrews (*Crocidura lasiura*) with IgG antibodies against Imjin virus (MJNV, a newly isolated hantavirus) had MJNV RNA detectable by RT-PCR [50]. In our study bats sampled in 2015 and 2016 showed higher seropositive rates, but HV RNA was not detected from either seropositive or seronegative bats. All nine RT-PCR positive samples were collected in between 2012–2014, but their antibody titers were not tested since the sera were not collected during that time. Moreover, serology study on bat-borne HVs was not conducted in previous publications, therefore further study is needed to understand the dynamics of HV infection and its antibody response in bats.

Serological epidemiology is important to uncover the real situation of bat-borne HV prevalence, and is critical for eventual estimation of the potential risk of these viruses to public health. Since bat-borne HVs have never been isolated, their NP is a preferential target for serological investigation and antigenic differentiation. It is the main immunogenic protein which contains both serotype-specific and group common epitopes, and is commonly used as diagnostic antigen for HV detection [22–25, 51]. For these reasons, rNPs of LAIV, XSV and SEOV expressed in *E*. *coli* were used to assay all 709 bat serum samples by ELISA, resulting in identification of a large number of seropositive sera (Fig 3), with many likely to cross react with two or three rNPs (see OD_492_ values in S2 Fig). To confirm this, 88 ELISA sera were further tested by WB against all three rNPs with results showing that, except for sera reacting exclusively with one rNP, some could cross recognize two rNPs, mainly the rNPs of LAIV/XSV, or XSV/SEOV, and seldomly the rNPs of LAIV/SEOV (see S3 Fig). It is notable that 21 sera could cross recognize three rNPs with 10 showing very strong reactivity against all three rNPs. The role of NP in producing this cross antigenicity was further verified by WB using a combination of eukaryotically expressed rNPs and NP-specific antiserum (see Fig 2B). To identify NAb against SEOV, 48 bat sera were analyzed by FAVNT, which identified 9 (18.8%) positives. Fig 4 summarizes the results of 48 bat sera assayed by FAVNT, ELISA and WB. Of the nine NAb-positive sera, four (BN5, ZS7, ZS25, ZS27) neutralized but did not react by ELISA or WB, three (BN64, CZ67, NP6) not only neutralized but also reacted with three rNPs by ELISA and WB. The most interesting bat serum was BN78, which neutralized SEOV and reacted with the rNP of SEOV but not with that of LAIV and XSV. BN78 was collected from a *Rousettus leschenaultii* bat, of this species 51 of 142 individuals showed anti-HV antibody positive (35.9%), the highest among all bat species (Table 1). Furthermore another Rousettus species (*Rousettus amplexicaudatus*) was reported to harbor Quezon virus in the Philippines [12], suggesting that fruit bats in genus *Rousettus* are likely major reservoirs of HVs. Moreover many sera without neutralizing activity reacted with the three rNPs by ELISA and WB. Altogether, it is interesting to have found multiple patterns of cross-reactivity with three rNPs. Illustration of the complex patterns will be difficult but likely to imply that the bats had been infected with other unknown HVs. The prime example is bat serum BN78. It had the highest neutralization titer against SEOV and exclusively strong reactivity with the rNP of SEOV, indicating that this bat was infected by an unknown HV antigenically very close to SEOV, but not SEOV since both human anti-SEOV convalescent serum (Fig 2A) and SEOV-specific anti-rNP serum (Fig 2B) could also cross react with the rNP of XSV. Altogether, the multiple genetic diversities and different cross-reactivity patterns indicate that more as yet unknown bat-borne HVs circulate in the investigated region, but to uncover them further investigation is needed.

### Host range of bat-borne HVs

Viruses usually have a defined host range for circulation in nature. It is interesting to note that LAIV BT33 in the present study and LAIV BT20 in a previous study [9] have been identified in different locations but from the same bat species (*Ta*. *melanopogon*). All XSV variants in the present and previous studies [7, 8] have come from a single bat species, *Hipposideros* (*Hi*. *pomona)*. Apart from MAKV in Gabon, which also came from a *Hipposideros sp*. (*Hi*. *ruber*), all other six HVs came from bats in six different genera. The host range of currently identified bat-borne HVs is summarized in Table 2. In general, bat-borne HVs have a huge genetic diversity with different viral species harbored by different genera of bats, showing wide range of hosts. But regarding a given bat-borne HV species its host range may be narrow, restricted mainly to one bat genus. This implies that a given bat-borne HV may have a host tropism.

### Circulation sphere of bat-borne HVs

As shown in Fig 5, 12 of the 13 cities in 4 provinces in which serum collections were made had a positive seroprevalence, with Guangxi having the most positive samples and most seropositive locations (6 of 7 sampled cities were seropositive). Furthermore, two HVs were detected in two of its cities, BS and LB. In 2015, LAIV was identified in LB [9], in which XSV was found in the present study although from another location within the city, indicating that divergent bat-borne HVs co-exist in LB. LAIV was also found in BS this time, several hundred kilometers west of LB (see Fig 5), indicating that LAIV has a broad distribution in Guangxi province. It is notable that XSV has been identified in two north Vietnamese provinces, Tuyên Quang (TQ) and Phú Thọ (PT), as shown in Fig 5, and in the central Vietnamese province Quảng Nam since 2013 [7, 8]. In present study eight strains of XSVs were identified in LB of Guangxi and PE of Yunnan, indicating that XSV circulates in the vast area between Chinese Guangxi/Yunnan and Vietnam. The accumulated serological and molecular data highly support the proposition that a vast area between China and southeast Asia provides a natural focus for bat-borne HV circulation. In this area natural circulation of genetically divergent bat-borne HVs in their hosts would be maintained, and therefore the concept of a bat-borne HV circulation sphere has been introduced to describe the situation. While there is a lack of sufficient serological data in Yunnan Province, a narrow area between southwest Guangxi and north Vietnam likely forms a main circulation sphere of at least two species of bat-borne HVs (Fig 5). With more extensive investigations this area may be extended, particularly to surrounding areas in Laos, Myanmar and even Thailand.

In conclusion, the present study has compiled the first profiling of cross antigenicity between bat-borne and human-infecting HVs as well as among bat-borne HVs. It has also revealed the seroprevalence and wide distribution of bat-borne HVs in south and southwest China. A comprehensive analysis based on genetic diversity, seroprevalence, cross antigenicity and host range of the viruses has helped identify an area between China and Vietnam as a main circulation sphere where at least two bat-borne HVs circulate in the bat population. Given the existence of bat-borne HVs genetically and antigenically close to human-infecting HVs, extensive studies should be emphasized in future to assess the potential risk of bat-borne HVs to public health.

## Materials and methods

### Ethics statement

The procedures for sampling bats and mouse experiments in this study were reviewed and approved by the Administrative Committee on Animal Welfare of the Institute of Military Veterinary Medicine (Laboratory Animal Care and Use Committee Authorization permit JSY-DW-2010-02 for bats and JSY-DW-2015-02 for mice). All live animals were maintained and handled according to the Principles and Guidelines for Laboratory Animal Medicine (2006), Ministry of Science and Technology, China.

### Sample information and metagenomic preparation

The 1,419 tissues (lungs and intestines) and 709 sera of 1,561 bats used in present study were archived and sub-packed samples stored at -80ºC following collection between 2012 and 2016 in 22 cities of Yunnan, Guangdong, Fujian, Zhejiang and Guangxi provinces, China, and used to investigate viruses than hantaviruses in our previous studies [32, 38, 52, 53]. Bat species were identified morphologically and then molecularly by sequencing the bat mitochondrial cytochrome *b* gene from muscle tissue [54]. These bats were classified as belonging to 22 species within 9 genera and 6 families: *Hipposideridae* (n = 598), *Rhinolophidae* (n = 358), *Vespertilionidae* (n = 172), *Miniopteridae* (n = 129), *Emballonuridae* (n = 74) and *Pteropodidae* (n = 230). Detailed sample information is shown in Fig 5 and Table 1. The tissue samples were homogenized and subjected to RNA extraction using the RNeasy Mini Kit (QIAGEN), and the RNA was reversely transcribed into cDNA which was processed for viral metagenomic analysis as described previously [55]. Serum samples were used in serological analyses.

### Hantaviral RNA detection

Identified HV-like contigs were subjected to BLASTn and BLASTx search (https://blast.ncbi.nlm.nih.gov/Blast.cgi). The genomic positions of the contigs were decided using Hantaan virus strain 76–118 as the reference. RNA was extracted from bat tissues (intestines with contents and lungs) and screened by RT-PCR using pan-HVs nested primers targeting a 396-bp sequence of the conserved L segment [40]. Positive amplicons were sent for Sanger sequencing (Comate) and the obtained sequences were used to initially determine their phylogenetic locations. Details of the primers can be seen in S2 Table.

### Virus isolation

HV positive lung tissues (~100 mg/bat) were thoroughly ground with DMEM (500 μL/100 mg tissue) in a homogenizer and clarified by centrifugation at 5,000 g for 5 min. Following sterilization by passage through a 0.22 μm Millipore filter supernatants were incubated with BHK-21 cells as well as the HV-sensitive cell lines Vero and Vero E6 [56] (all stored in our laboratory) in 24-well plates. After incubation for 24 h at 37ºC, the cells were washed 2x with PBS, incubated in DMEM with 2% FBS for 14–21 days and observed daily for cytopathic effects (CPE). Cultures, if showing no CPE, were harvested by freeze-thawing 3x and passaged again in the same cell lines. After five passages, the cultures were analyzed for HV by RT-PCR.

### Whole genome sequencing and comparison

To characterize the full genomic sequence and structure of detected HVs, genome-amplifying overlapping primers were synthesized based on the contigs (S1 Table) and representative sequences of previously published HVs including bat-borne HV LAIV BT20 [9]. Since the terminal nucleotide sequences of S, M and L segment are conserved among members of the genus *Orthohantavirus* [1], their sequences were used as primers to obtain the end sequences of each segment. The targeting amplicons were amplified using the Phusion High-Fidelity PCR Master Mix with HF Buffer (NEB) with the recommend reaction system, and cloned into a blunt-end pLB vector using a Lethal Based Fast Cloning Kit (Tiangen). Three clones of each amplicon were further identified by PCR and then sent for commercial Sanger sequencing. The complete genomic sequences were obtained by assembling amplicons with overlapped regions using SeqMan in the DNAStar software package. ORFs of each segment were searched by ORFfinder (https://www.ncbi.nlm.nih.gov/orffinder/) in NCBI and the predicted proteins were further confirmed by aligning in BLASTp.

### Phylogenetic analyses

The representative sequences of each classified HV species as well as some unclassified HVs were downloaded from GenBank. Their complete NP, GP and RdRp coding regions (aa) were aligned with those obtained in the present study using the online program MAFFT version 7 (https://mafft.cbrc.jp/alignment/server/). The best-fit substitution model for each tree was selected based on the Akaike Information Criterion (AIC) of Smart Model Selection (SMS) in PhyML (version 3.0) [57]. Phylogenetic trees, including their topology and support for tree nodes, were then inferred using the maximum likelihood method, Subtree Pruning and Regrafting (SPR), approximate Likelihood Ratio Test (aLRT) with the Shimodaira-Hasegawa-like (SH-like) tree-selection method in PhyML [58]. Sequence identities were calculated by MegAlign in DNAStar software package.

### rNP expression in *E*. *coli* and generation of mouse anti-rNP specific hyperimmune serum

The complete NP coding sequences (CDS) of bat-borne HV LAIV, XSV strains identified in the present study were amplified with the 5’ *Eco*RI and a 3’ *Xho*I sites at the two ends. The complete CDS of SEOV strain GuangzhouRn36 (1,287 nt, Accession number: GU592948) was optimized using *E*. *coli* codons and chemically synthesized (GENEWIZ) with the same restriction enzyme sites (primers shown in S2 Table). The NP gene fragments so obtained were subcloned into a prokaryotic expression vector pET-28a(+) with a His-Tag at C terminus and used to transfected *E*. *coli* strain Rosetta (Tiangen). The rNPs were expressed after induction with 0.5 mM isopropyl-β-d-thiogalactoside (IPTG) and identified by SDS-PAGE and WB using mouse anti-6X His-tag monoclonal antibody and Alexa Fluor 680-conjugated donkey anti-mouse IgG H&L (Abcam) as the respective primary and secondary antibodies. The rNPs of the three HVs were purified and quantified by Ni-NTA His Bind Resin (Novagen) and BCA Protein Assay Kit (CWBio), following which they were identified using an SEOV-convalescent human serum (H anti-SEOV) and a negative human serum (H Neg.) as controls (both stored in our laboratory). The H anti-SEOV serum was collected from a SEOV-infected convalescent patient who was diagnosed at onset by clinical symptoms and RT-PCR.

Specific hyperimmune sera were prepared by injecting four week-old female Kunming mice intramuscularly with purified rNPs of the three HVs. Each injection contained 20 μg protein mixed 1:1 (V/V) with the Quick Antibody-Mouse 5W adjuvant (Biodragon) as recommended by the producers and booster doses with the same formulated rNP were given at 14 days later. At 21 days post boost, blood was collected through heart puncture for serum preparation. Antibody titers were determined by rNP-based ELISA as described below.

### rNP expression in mammalian cells

Complete CDS of NPs of the same LAIV, XSV and SEOV strains were amplified with primers containing a 5’ *Xho*I site and a 3’ *Eco*RI site. Three NP fragments were fused in-frame to the C-terminal of the enhanced green fluorescent protein (EGFP) tag (239 aa, MW 27 kDa) of eukaryotic expression vector pEGFP-C1. Transient expressions of rNPs were obtained by transfection of BHK-21 cells in 6-well cell plates with the constructed plasmids with the blank vector pEGFP-C1 as control, using Lipofectamine2000 reagent (Invitrogen) according to the manufacturer’s protocol. The transfected cells were cultured at 37ºC with 5% CO_2_ for 36 h, and then examined microscopically for the expression of fusion proteins with green fluorescence. NP-expressing cell cultures were collected and lysed with Cell Lysis Buffer (CST), and the total protein was quantified using the BCA Protein Assay Kit. Correct expression of rNPs in cell lysates were confirmed by WB using mouse anti-EGFP monoclonal antibody (Abcam) and Alexa Fluor 680-conjugated donkey anti-mouse IgG H&L as the respective primary and secondary antibodies.

### Analysis of cross-reactivity by WB

Eukaryotically expressed NPs were used as antigens to detect antibodies in mouse hyperimmune sera by WB. Briefly the eukaryotically expressed NPs were separated by SDS-PAGE and transferred onto nitrocellulose blotting membranes (GE Healthcare), blocked with 5% non-fat milk (Promega) at 4ºC overnight, then incubated with the above three mouse anti-NP hyperimmune sera at 1:300 dilution for 2 h. After washing with PBST 3x, the membranes were incubated with Alexa Fluor 680-conjugated donkey anti-mouse IgG H&L 1:1,000 for 50 min. After washing, the membranes were scanned and photographed using an Odyssey imager (LI-COR Biosciences). All WB analyses were repeated at least three times.

### Serological assay of bat sera by ELISA and WB

ELISAs using rNPs as coating antigen were developed to detect antibodies of bat against LAIV, XSV and SEOV. Briefly 96-well microplates (Corning) were coated with purified prokaryotically-expressed rNPs (200 ng/well in NaHCO_3_-Na_2_CO_3_ buffer at pH 9.6) at 4°C overnight and blocked with 5% non-fat milk (Promega) at 37°C for 1 h. Then 100-fold PBS-diluted serum samples were added to the wells (duplicate wells per serum) and incubated at 37°C for 1 h followed by addition of HRP-conjugated goat anti-bat IgG, H&L chain, polyclonal antibody (BETHYL, react specifically with bat IgG, and with light chains common to other bat immunoglobulins) at a 1:20,000 dilution for bat sera, and HRP-conjugated goat anti-human IgG polyclonal antibody (Zsbio) at a 1:200 dilution for human sera (controls). After incubation at 37ºC for 50 min, freshly-prepared O-phenylenediamine (OPD) substrate solution (Sigma) was added to each well for 5 min for color reaction, which was stopped by addition of 2 M sulfuric acid. The OD_492_ values were immediately read and blanked by the OD_630_ value using a Multimode Microplate Reader (Infinite 200 PRO, Tecan). The cut-off values of bat sera were determined based on the highest CR between results of ELISA and WB.

To validate the ELISA result, WBs using prokaryotically expressed rNPs were performed to detect bat and human sera. Briefly, the rNPs were separated by SDS-PAGE, transferred onto nitrocellulose membranes followed by blocking using the protocol described above. Membranes were then incubated with 1:300 diluted selected bat or human serum for 2 h, followed by 2,000-fold diluted HRP-conjugated goat anti-bat IgG H&L chain polyclonal antibody or 100-fold diluted HRP-conjugated goat anti-human IgG polyclonal antibody for 50 min. All procedures were conducted at ambient temperatures. Bands were pictured by automatic chemiluminescence (Tanon).

### FAVNT

To determine the NAb titers of bat and human sera, the FAVNT using 200 TCID_50_ SEOV (10^5.31^TCID_50_/0.1mL) in Vero E6 cells was performed by a previously published protocol [59]. Anti-HV monoclonal antibody provided by the Fourth Military Medical University [60] was labeled using FITC [61]. The NAb titer of each serum was calculated using the Spearman-Kärber formula [62].

### Statistics

To compare the differences of the OD_492_ values of bat and human sera to each HV, normal distribution tests were conducted separately, and multiple comparison was performed using t tests (LSD), Student-Newman-Keuls (SNK) test, Tukey's studentized range (HSD) test, Bonferroni (Dunn) t tests and Scheffe's test. Then κ test, an inter-rater agreement statistic, was used to evaluate the consistency between the results of ELISA and WB. All statistics were programed and calculated using the Statistical Analysis System (SAS) version 9.2.

## Supporting information

S1 Fig The sketched contig locations in hantavirus genome. The reference M (A) and L (B) sequences are of HTNV strain 76–118 (Accession number: Y00386 and NC_005222). Red: LAIV-like contigs; Blue: XSV-like contigs. Length of arrow represents the length of the contig.
(TIF)

S2 Fig The OD_492_ values of 709 bat sera against the rNPs of LAIV, XSV and SEOV. Each bar on the X axis represents 1 of the 709 serum samples and their geographical source, YN: Yunnan; GX: Guangxi; FJ: Fujian; ZJ: Zhejiang.
(TIF)

S3 Fig WB assay of 88 bat sera in 4 provinces. Sampling provinces: (A) Yunnan, (B) Guangxi, (C) Fujian, (D) Zhejiang. OD_492_ value is below each lane with positive reading in bold. 0.2 μg/lane rNP of LAIV, XSV and SEOV were separately loaded from left to right.
(TIF)

S4 Fig The coincidence rate (CR) between ELISA and WB at different proposed cut-off values. The cut-off values finally determined for ELISA (0.10 for LAIV and XSV, and 0.11 for SEOV) with the correspondent CR (87.5%, 86.4% and 86.4%) are marked.
(TIF)

S5 Fig Phylogenetic tree generated based on the 314-bp sequence of the L segment using the method of Fig 1. Bootstrap values of 1,000 replicates (>0.7) are shown and the scale bars indicate nucleotide substitutions per site. Red: bat-borne HVs, blue: insectivore-borne HVs, green: rodent-borne HVs, filled red circles: sequences obtained in this study. Definitions of virus abbreviations and their GenBank accession numbers are in S4 Table.
(TIF)

S1 Table The information of contigs annotated to HVs obtained from high-throughput sequencing. (DOC)

S2 Table Primers designed for sequence amplification of HVs. (DOC)

S3 Table The nt/aa identities of the complete genomic sequences obtained in the present study compared with those of rodent- and insectivore-borne HVs. (DOC)

S4 Table Information of reference sequences used in the present study. (DOC)
