# Supplementary figures and images for: Seroprevalence, cross antigenicity and circulation sphere of bat-borne hantaviruses revealed by serological and antigenic analyses
Source: PLoS Pathog. 2019 Jan 22;15(1):e1007545. doi: 10.1371/journal.ppat.1007545 (PMC6358112; doi:10.1371/journal.ppat.1007545)

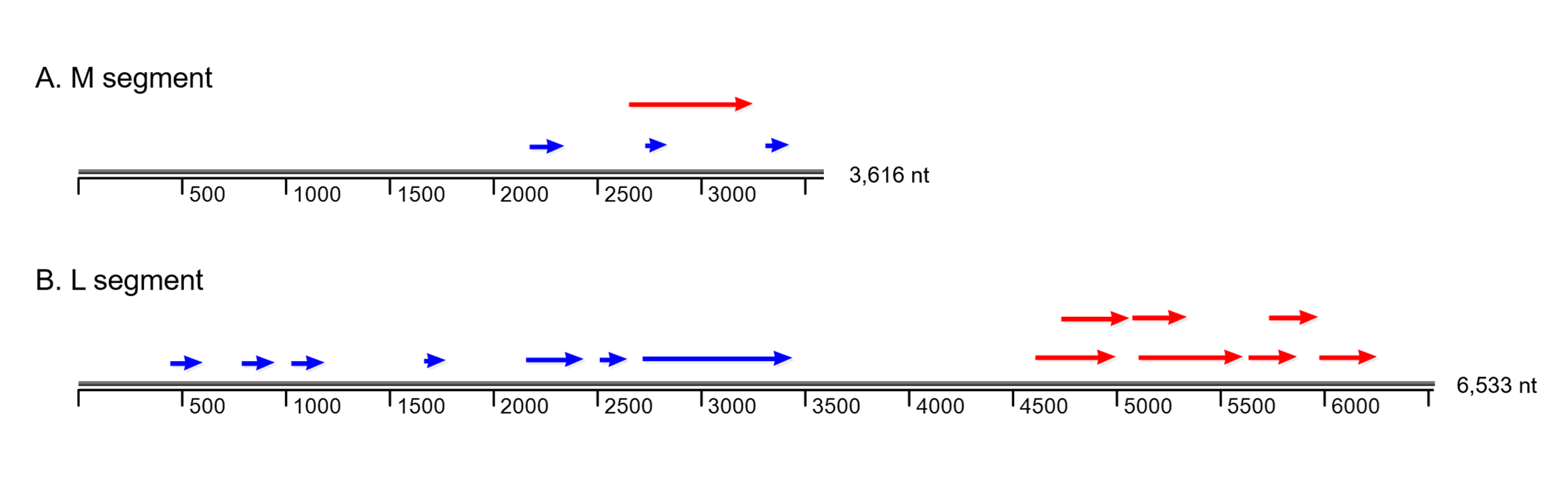

Supplement: S1 Fig — The reference M (A) and L (B) sequences are of HTNV strain 76–118 (Accession number: Y00386 and NC_005222). Red: LAIV-like contigs; Blue: XSV-like contigs. Length of arrow represents the length of the contig. (TIF) [file ppat.1007545.s001.tif]

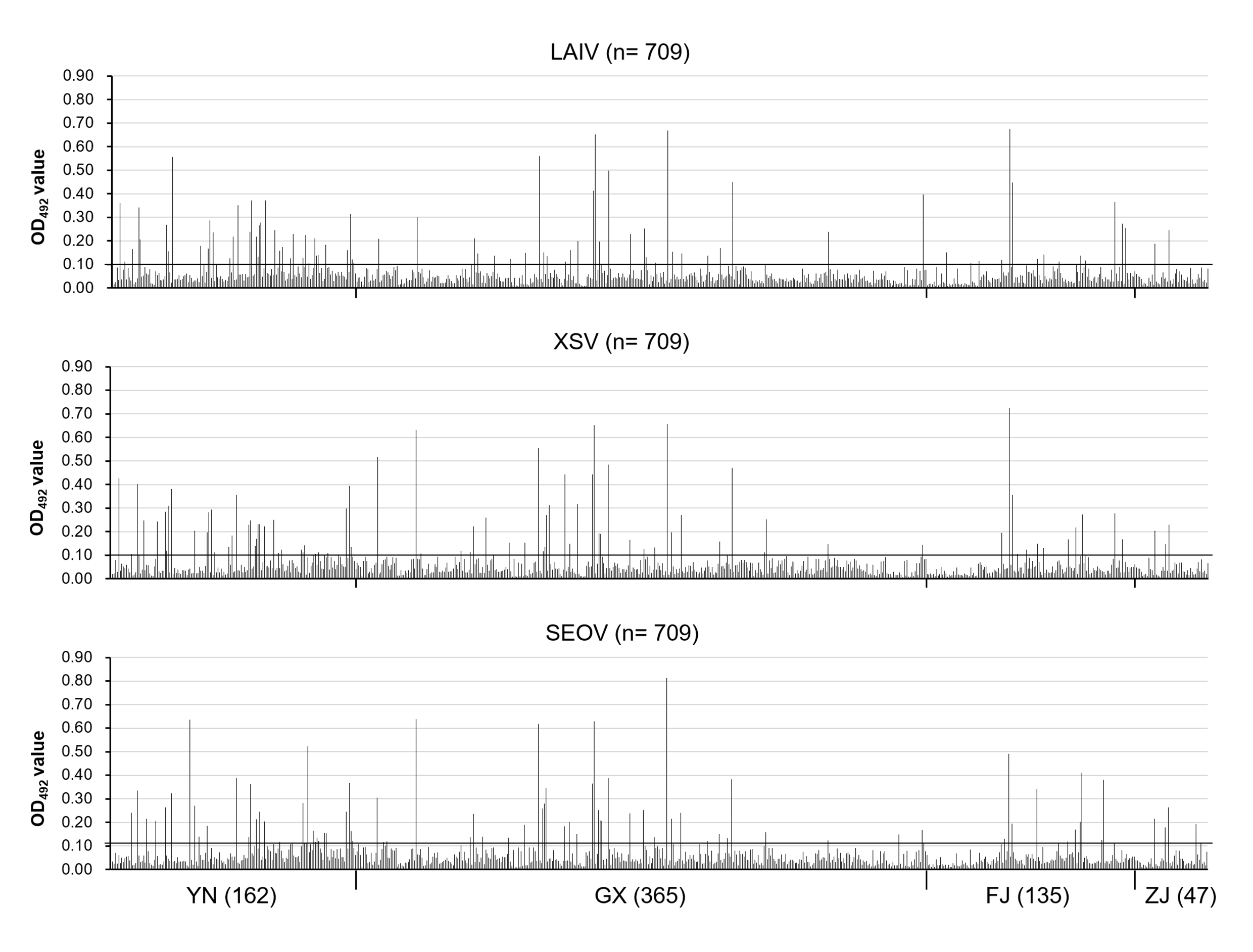

Supplement: S2 Fig — Each bar on the X axis represents 1 of the 709 serum samples and their geographical source, YN: Yunnan; GX: Guangxi; FJ: Fujian; ZJ: Zhejiang. (TIF) [file ppat.1007545.s002.tif]

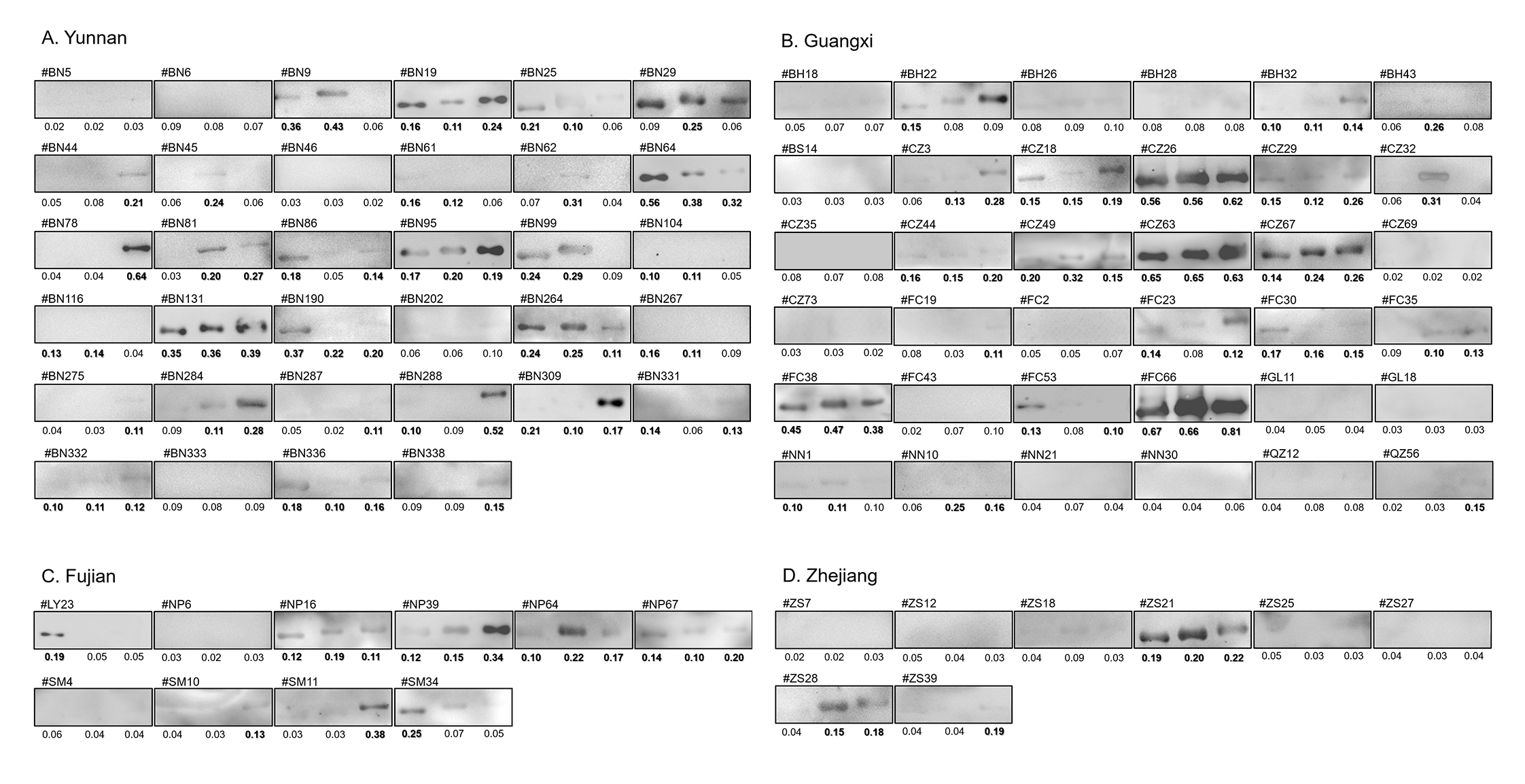

Supplement: S3 Fig — Sampling provinces: (A) Yunnan, (B) Guangxi, (C) Fujian, (D) Zhejiang. OD492 value is below each lane with positive reading in bold. 0.2 μg/lane rNP of LAIV, XSV and SEOV were separately loaded from left to right. (TIF) [file ppat.1007545.s003.tif]

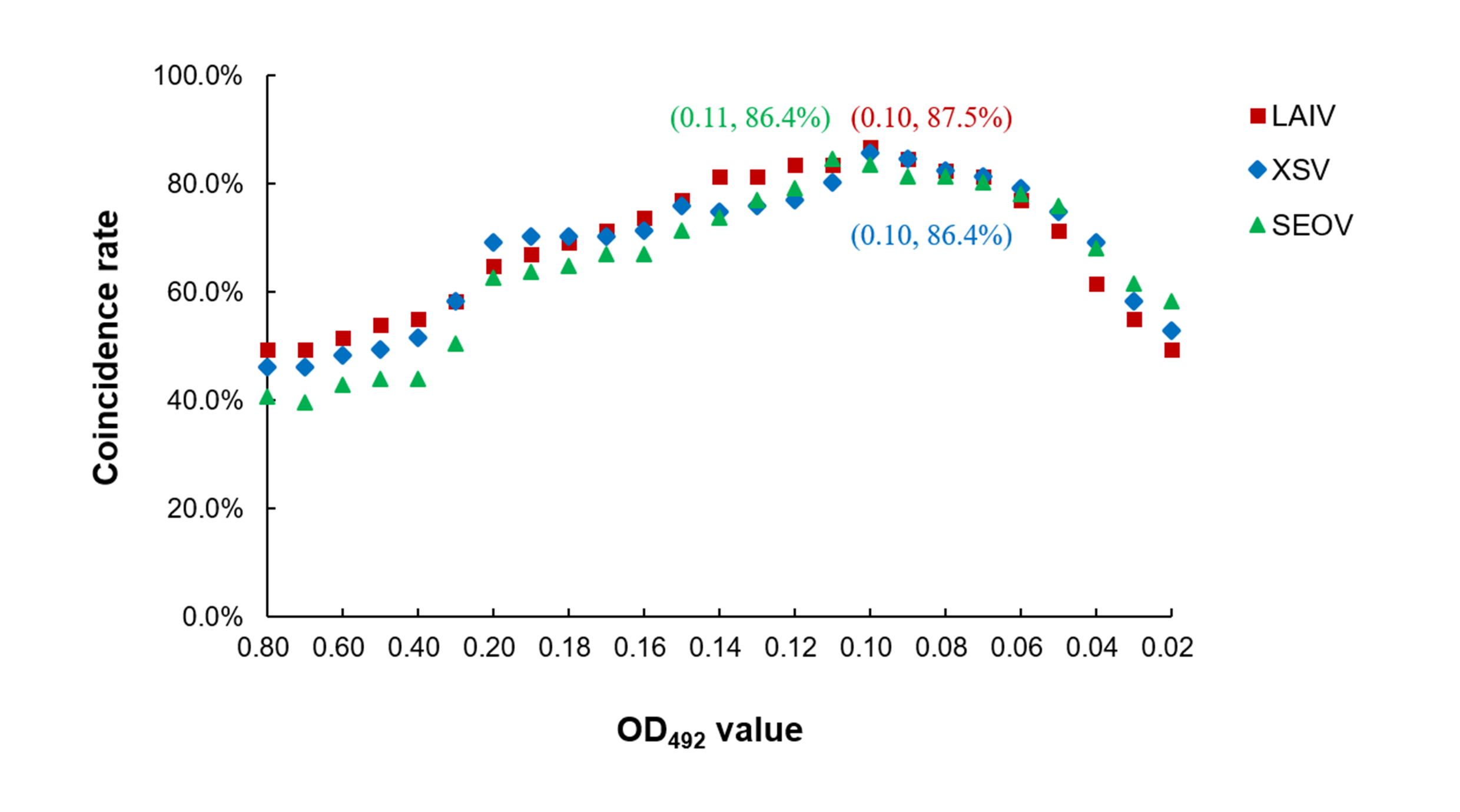

Supplement: S4 Fig — The cut-off values finally determined for ELISA (0.10 for LAIV and XSV, and 0.11 for SEOV) with the correspondent CR (87.5%, 86.4% and 86.4%) are marked. (TIF) [file ppat.1007545.s004.tif]

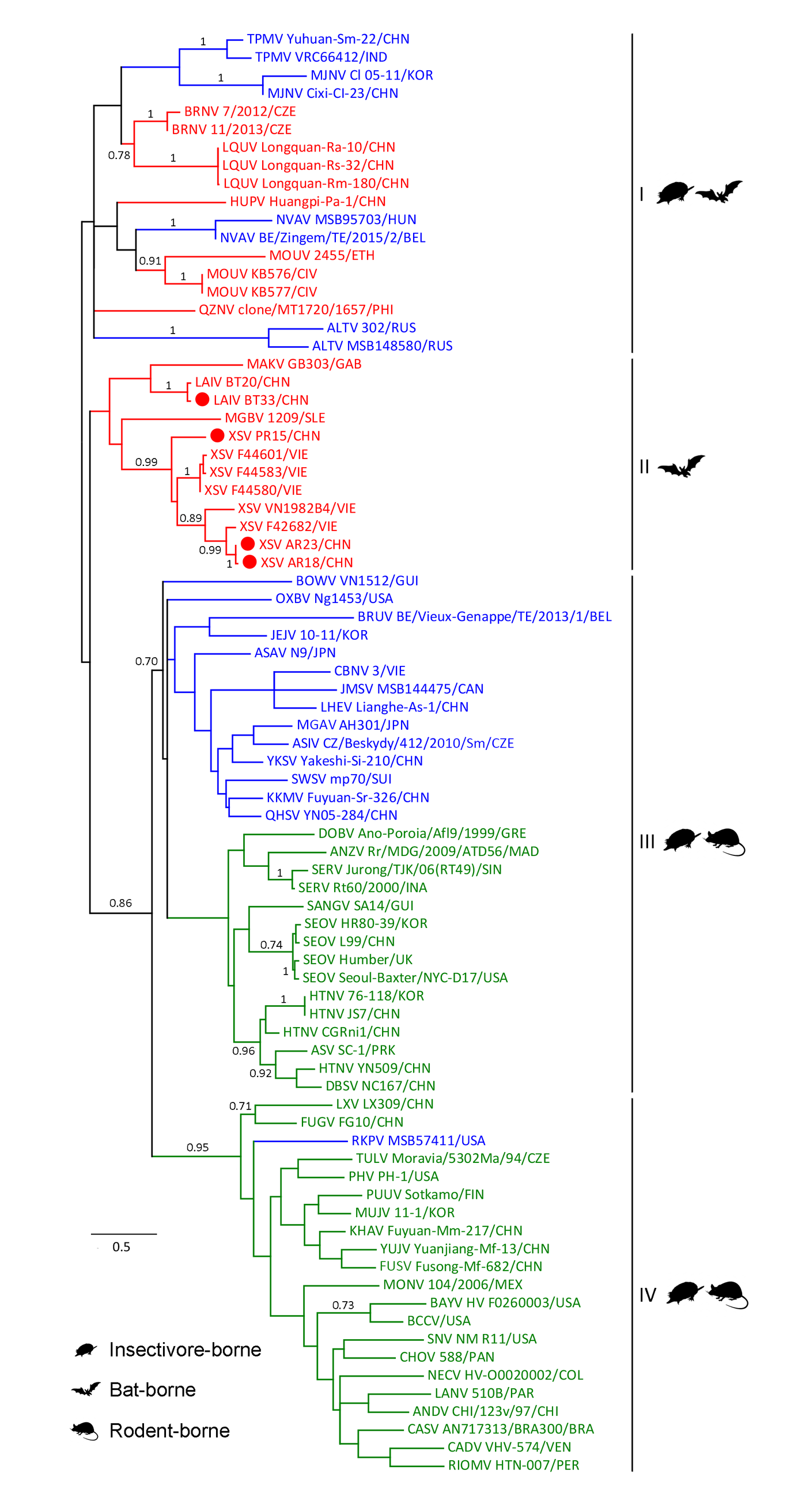

Supplement: S5 Fig — Bootstrap values of 1,000 replicates (>0.7) are shown and the scale bars indicate nucleotide substitutions per site. Red: bat-borne HVs, blue: insectivore-borne HVs, green: rodent-borne HVs, filled red circles: sequences obtained in this study. Definitions of virus abbreviations and their GenBank accession numbers are in S4 Table. (TIF) [file ppat.1007545.s005.tif]
