# Supplementary material for: Seroprevalence, cross antigenicity and circulation sphere of bat-borne hantaviruses revealed by serological and antigenic analyses
Source: PLoS Pathog. 2019 Jan 22;15(1):e1007545. doi: 10.1371/journal.ppat.1007545 (PMC6358112; doi:10.1371/journal.ppat.1007545)
Supplement: S1 Table — (DOC) [file ppat.1007545.s006.doc]

**S1 Table. The information of contigs annotated to HVs obtained from high-throughput sequencing.**

| Location | Contigs | | Blast | | |
| --- | --- | --- | --- | --- | --- |
|  | NO. | Length (bp) | Hantavirus | Segment | Identity % (nt/aa) |
| Baise,  Guangxi | 3 | 243-330 | LAIV | L | 98-99/100 |
|  | 1 | 594 | LAIV | M | 99/99 |
| Laibin,  Guangxi | 7 | 110-726 | XSV | L | 82-90/84-100 |
|  | 4 | 228-503 | LAIV | L | 72-77/84-89 |
|  | 2 | 110-120 | XSV | M | 83-86/94-98 |
| Puer,  Yunnan | 1 | 171 | XSV | M | 87/98 |
