# Supplementary material for: Seroprevalence, cross antigenicity and circulation sphere of bat-borne hantaviruses revealed by serological and antigenic analyses
Source: PLoS Pathog. 2019 Jan 22;15(1):e1007545. doi: 10.1371/journal.ppat.1007545 (PMC6358112; doi:10.1371/journal.ppat.1007545)
Supplement: S2 Table — (DOC) [file ppat.1007545.s007.doc]

**S2 Table. Primers designed for sequence amplification of HVs.**

| Virus | Primers | Sequence (5’-3’) | Polarity | Source |
| --- | --- | --- | --- | --- |
| LAIV | *Detection* | | | |
|  | HAN-L-F1 | ATGTAYGTBAGTGCWGATGC | + | Ref. [33] |
|  | HAN-L-R1 | AACCADTCWGTYCCRTCATC | - |  |
|  | HAN-L-F2 | TGCWGATGCHACNAARTGGTC | + |  |
|  | HAN-L-R2 | GCRTCRTCWGARTGRTGDGCAA | - |  |
|  | *Genomic amplification* | | |  |
|  | SF1 | TAGTAGTAGACTCCTTRAARAGC | + | Ref. [7] |
|  | SR1071 | AGCTCNGGATCCATITCATC | - |  |
|  | SF1283 | CAAACTAGTAGGAACTGCTGA | + |  |
|  | SR1906 | TAGTAGTAKRCWCCYTRARAAG | - |  |
|  | MF1 | TAGTAGTAGACWCCGCAAAAG | + |  |
|  | MR1368 | CTTTTCTTTGCCCATTG | - |  |
|  | MF1150 | GGACCAGGTGCADCTTGTGAAGC | + |  |
|  | MR2611 | CATGAYATCTCCAGGGTCHCC | - |  |
|  | MF2517 | GTAGTAACCATGCTAAGGTATG | + |  |
|  | MR3684 | TAGTAGTATRCTCCGCARG | - |  |
|  | LF1 | TAGTAGTAGACTCCRGA | + |  |
|  | LR400 | GTGTTTTACCTGTAGGGT | - |  |
|  | LF186 | ATGATAAARCATGAYTGGTC | + |  |
|  | LR2260 | TGDATYTTDGCTTCTTCWGTCA | - |  |
|  | LF1959 | GCTAGTTAYTCMGGTGCTGC | + |  |
|  | LR3393 | AACCADTCWGTYCCRTCATC | - |  |
|  | LF3341 | TTGCHCAYCAYTCWGAYGATGC | + |  |
|  | LR5500 | CTCTTTAGTCACCTCTTCCAT | - |  |
|  | LF5178 | TCWGGDAARCARTATGATGC |  |  |
|  | LR6561 | TAGTAGTAGTAKRCTCCGRGA | + |  |
|  | *NP expression* | | |  |
|  | LAIV-NF | ccgGAATTCATGGCTTCAATAGCAGATCTCC ^b^ | + |  |
|  | LAIV-NR | ccgCTCGAGTTAAATTCTAAGAGGTTCTTGG | - |  |
|  | LAIV-SF | ccgCTCGAGCTATGGCTTCAATAGCAGAT | + |  |
|  | LAIV-SR | ccgGAATTCTTAAATTCTAAGAGGTTCTTGG | - |  |
| XSV | *Genomic amplification* ^a^ | | | |
|  | XSF0 | CTCGAATTCTAGTAGTAGRCTCC | + | This study |
|  | XSR1235 | GTTCTTCTGAGATATGACTGATA | - |  |
|  | XSF988 | ATAGATGTCCWCCTACAGCAGT | + |  |
|  | XSR1752 | CCTAGTAGTATDCTCCYTARTAAGC | - |  |
|  | XMR1657 | CTTACAACTACACTCTCTGTGAC | - |  |
|  | XMF2357 | TTTCTAGATAAGATCACACC | + |  |
|  | XLR2364 | GCTGTTCAACTATAACTG | - |  |
|  | XLF3174 | CAGATGGGAATTCTGGCTTGGTTCGA | + |  |
|  | XLR5715 | TATTCCTTTCATGAAGTTCTCTA | - |  |
|  | XLF5578 | GATTTGGAGAGTCCTAGATTTC | + |  |
|  | XLR6182 | CATCAAGATTATACCTATTGGGT | - |  |
|  | XLF6183 | CCCAATAGGTATAATCTTGATG |  |  |
|  | XM-R2242 | TTACATTCHCCATAACARTGAAA | - |  |
|  | XM-F2165 | ATATCTTGGGCAYTGGATGGA | + |  |
|  | XL-F0 | TAGTAGTAGGACTCCGGATAC | + |  |
|  | XL-R2260 | TTGTGCCCAYTCAACAGTCTC | - |  |
|  | XL-F3266 | AAAGGGTATGGAATGAATTGTA | + |  |
|  | XL-R6223 | TGCTGTTCACCAAAATTRTAATG | - |  |
|  | *NP expression* | | |  |
|  | XSV-NF | ccgGAATTCATGGCAACACAGGCGGATCT | + |  |
|  | XSV-NR | ccgCTCGAGTTAAAGCTTTAGTGGTTCTTGG | - |  |
|  | XSV-SF | ccgCTCGAGCTATGGCAACACAGGCGGAT | + |  |
|  | XSV-SR | ccgGAATTCTTAAAGCTTTAGTGGTTCTTGG | - |  |
| SEOV | *NP expression* | | |  |
|  | SEOV-SF | ccgCTCGAGCTATGGCAACCATGGAAGAGAT | + |  |
|  | SEOV-SR | ccgGAATTCTTACAGTTTCATAGGTTCCTGG | - |  |

^a^ Some primers of LAIV were used to amplify the complete genome of XSV strains.

^b^ Protective base were shown in lowercase letters and restriction enzyme cutting sits were underlined.
