# Supplementary material for: Seroprevalence, cross antigenicity and circulation sphere of bat-borne hantaviruses revealed by serological and antigenic analyses
Source: PLoS Pathog. 2019 Jan 22;15(1):e1007545. doi: 10.1371/journal.ppat.1007545 (PMC6358112; doi:10.1371/journal.ppat.1007545)
Supplement: S3 Table — (DOC) [file ppat.1007545.s008.doc]

**S3 Table. The nt/aa identities of the complete genomic sequences obtained in present study with those of rodent- and insectivore-borne HVs. ^a^**

| Host | Virus | LAIVBT33 | | | XSVAR18 | | | XSVPR15 | | |
| --- | --- | --- | --- | --- | --- | --- | --- | --- | --- | --- |
|  |  | S/NP | M/GP | L/RdRP | S/NP | M/GP | L/RdRP | S/NP | M/GP | L/RdRP |
| Rodent | ANDV | 53.4/54.4 | 49.6/47.3 | 63.7/64.1 | 53.7/55.6 | 53.4/47.3 | 63.7/64.2 | 54.8/55.8 | 52.5/46 | 63.8/64.5 |
|  | BAYV | 53.9/54.2 | 49.8/47.7 | 63.3/64.5 | 49.6/54.7 | 52.7/47.3 | 63.5/64.7 | 51.3/55.6 | 52.1/45.9 | 63.1/64.8 |
|  | BCCV | 52.8/54.4 | 49.4/47.2 | - | 50.8/54.9 | 53.5/46.7 | - | 51.2/55.6 | 52.2/46 | -/- |
|  | CADV | 53.9/53.3 | 50.0/48.5 | 64.2/64.5 | 49.2/53.8 | 53.5/48.9 | 63.6/64.5 | 50.4/53.8 | 52.3/47.2 | 63.6/64.9 |
|  | CHOV | 53.7/55.1 | 49.6/48.9 | 63.2/64.3 | 50.1/56.4 | 53.4/48.1 | 63.7/64.3 | 49.3/57.3 | 52.6/46.7 | 62.8/64.6 |
|  | DBSV | 48.3/52.0 | 48.7/44.9 | 63.3/64.0 | 57.7/52.7 | 51.3/45 | 62.9/64.4 | 57.1/52.9 | 50.9/43 | 63.7/64.7 |
|  | DOBV | 50.0/54.0 | 48.6/46.0 | 63.7/64.6 | 58.7/54.4 | 51.5/45.7 | 63.7/64.6 | 58.8/54.4 | 50.7/44.2 | 63.2/65.1 |
|  | ELMCV | 53.3/53.1 | 51.0/48.5 | - | 53.4/54.4 | 55.0/47.9 | - | 53.4/54.4 | 53.5/46.7 | -/- |
|  | FUGV | 53.5/53.8 | 50.0/48.1 | 64.3/64.1 | 59.0/54.4 | 53.1/47.0 | 64.1/63.9 | 58.6/54.4 | 52.2/46 | 63.5/64.1 |
|  | HTNV | 49.2/53.3 | 49.0/46.2 | 63.3/64.4 | 58.9/54.0 | 52.4/46.0 | 63.5/64.3 | 57.9/54 | 52.6/44.7 | 63.6/64.7 |
|  | KHAV | 52.6/54.7 | - | - | 56.9/55.6 | - | - | 55.5/56.2 | -/- | -/- |
|  | LANV | 53.3/54.0 | 49.5/47.4 | - | 54.2/54.9 | 53.8/47.3 | - | 55/55.6 | 51.9/46.4 | -/- |
|  | LXV | 48.5/53.6 | 49.7/48.1 | 63.3/63.6 | 45.3/53.1 | 53.8/47.3 | 63.5/63.7 | 43.7/53.1 | 52.3/46.2 | 63.4/64.3 |
|  | MONV | 50.7/54.2 | - | 63.3/64.7 | 47.5/54.0 | - | 63.8/65.2 | 47.5/54 | -/- | 63.3/65.5 |
|  | MPRLV | 53.9/54.4 | - | 63.4/64.1 | 54.6/54.2 | - | 63.1/64.7 | 54.1/54.2 | -/- | 63.4/64.9 |
|  | NECV | 52.7/53.1 | - | - | 48.7/53.8 | - | -/- | 49.2/53.8 | -/- | -/- |
|  | PHV | 50.2/52.9 | 49.4/47.3 | 63.1/64.2 | 59.0/54.0 | 53.9/46.8 | 62.8/63.7 | 59.3/54.9 | 52.3/45.8 | 62.3/64.2 |
|  | PUUV | 53.6/53.1 | 49.5/45.6 | 65.1/65.2 | 56.5/54.7 | 51.4/46.1 | 64.2/64.5 | 56.2/55.3 | 51.5/44.6 | 63.9/65.2 |
|  | SANGV | 51.6/53.3 | 48.6/46.0 | 62.9/64.1 | 60.0/53.3 | 51.9/45.7 | 63.8/64.8 | 59.0/53.8 | 51/44.5 | 63.1/65.4 |
|  | SEOV | 49.8/52.2 | 48.3/46.0 | 63.7/64.2 | 59.0/52.7 | 53.6/45.2 | 64.3/64.8 | 58.2/52.7 | 51.7/43.8 | 63.4/65.1 |
|  | SNV | 49.7/53.6 | 50.2/48.7 | 63.6/64.5 | 45.6/53.1 | 52.4/47.2 | 63.8/64.8 | 45.4/53.6 | 52/46.1 | 63.1/65 |
|  | THAIV | 53.1/54.0 | 48.9/46.0 | - | 54.7/54.0 | 53.4/45.7 | - | 55.1/54.0 | 52/44.5 | -/- |
|  | TULV | 53.2/54.7 | 50.3/47.8 | 62.9/64.3 | 56.9/55.3 | 51.7/46.4 | 63.3/63.9 | 55.8/55.6 | 52.5/46 | 63.2/64.3 |
|  | VLAV | 52.2/53.3 | - | - | 55.3/54.4 | - | - | 55.3/54.9 | -/- | -/- |
| Insectivore | AGMV | 47.5/53.3 | 48.7/44.5 | - | 57.0/51.1 | 52.1/44.2 | - | 56.0/50.2 | 52.9/42.7 | -/- |
|  | ASAV | 50.8/51.8 | - | - | 58.5/52.4 | - | - | 59.0/52.4 | -/- | -/- |
|  | ASIV | 46.1/52.9 | 49.0/44.8 | - | 55.0/50.9 | 51.5/44.7 | - | 55.2/50.2 | 51.7/43.3 | -/- |
|  | BOWV | 51.0/51.6 | 48.4/43.3 | 63.2/63.1 | 58.5/52.7 | 52.3/43.8 | 63.2/63.7 | 58.8/52.4 | 50.3/42.8 | 63.5/63.5 |
|  | BRUV | 53.0/54.0 | 49.0/46.6 | 63.9/64.3 | 56.1/54.7 | 52.5/44.9 | 64.1/64.2 | 55.9/54.9 | 52/44.3 | 64.1/64.6 |
|  | CBNV | 52.5/53.8 | 49.6/45.4 | 62.9/64.4 | 55.8/53.8 | 51.9/45.7 | 62.2/65.6 | 57.9/52.7 | 50.7/43.8 | 62/66 |
|  | JEJV | 51.8/52.7 | 48.2/45.0 | 63.3/63.2 | 59.9/52.7 | 52.1/45.2 | 63.2/63.4 | 61.2/52.7 | 50.7/44.1 | 64.1/63.5 |
|  | KKMV | 46.2/53.6 | 49.1/45.1 | 63.9/64.6 | 56.3/51.1 | 50.0/44.9 | 63.5/65.1 | 55.3/50.7 | 51/43.2 | 63.6/65.3 |
|  | MJNV | 43.4/46.0 | 45.9/41.4 | 67.1/64.4 | 53.0/47.1 | 61.8/40.3 | 66.7/64.0 | 54.3/46.9 | 48.9/39.8 | 66.8/64.3 |
|  | **NVAV** ^b^ | **54.0/60.2** | **57.3/58.8** | **66.6/68.4** | **57.8/59.3** | **51.8/58.7** | **66.1/67.5** | **58.9/59.1** | **58.8/57** | **66.2/67.6** |
|  | OXBV | 50.4/52.7 | 49.6/44.7 | - | 58.2/52.7 | 53.6/44.3 | - | 57.9/52.2 | 50.6/42.7 | -/- |
|  | RKPV | 55.4/55.8 | 50.3/48.2 | 64.0/63.4 | 57.3/55.1 | 49.4/47.3 | 63.6/63.7 | 57.5/55.1 | 52.2/46.2 | 63.8/64.3 |
|  | TPMV | 43.4/44.0 | 46.0/41.9 | 63.6/65.5 | 51.8/44.7 | 51.4/40.5 | 63.2/64.8 | 52.2/44.9 | 48.8/40 | 62.5/65.3 |
|  | YKSV | 48.6/53.8 | 48.5/44.4 | - | 59.0/51.6 | 65.8/43.9 | - | 57.6/55.1 | 51.1/42.9 | -/- |

^a^ Sequence information of the HVs was shown in Table S4. “-” means full-length sequence not available.

^b^ Insectivore-borne NVAV clustered closely to bat-borne HVs than to insectivore-borne HVs, showed the highest identities to the four strains and written in boldface.
