# Supplementary material for: Seroprevalence, cross antigenicity and circulation sphere of bat-borne hantaviruses revealed by serological and antigenic analyses
Source: PLoS Pathog. 2019 Jan 22;15(1):e1007545. doi: 10.1371/journal.ppat.1007545 (PMC6358112; doi:10.1371/journal.ppat.1007545)
Supplement: S4 Table — (DOC) [file ppat.1007545.s009.doc]

**S4 Table. Information of reference sequences used in present study.**

| Hantavirus strains | Abbreviation | Country | Collect year | Host | Accession NO. | | |
| --- | --- | --- | --- | --- | --- | --- | --- |
|  |  |  |  |  | S segment | M segment | L segment |
| Altai virus 302 | ALTV | Russia | 2007 | *Sorex araneus* | KP657656 | - | EU424341 |
| Altai virus MSB148580 | ALTV | Russia | 2010 | *Sorex caecutiens* | KM361044 | KM361051 | KM361056 |
| Amga virus AH301 | MGAV | Japan | 2010 | *Sorex caecutiens* | KF974360 | KF974359 | KF974361 |
| Andes virus Chile/123v/97 | ANDV | Chile | 1997 | *Oligoryzomys longicaudatus* | AF291702 | AF291703 | AF291704 |
| Anjozorobe virus Rr/MDG/2009/ATD56 | ANZV | Madagascar | 2008 | *Rattus rattus* | KC490916 | KC490921 | KC490923 |
| Asama virus N9 | ASAV | Japan | 2008 | *Urotrichus talpoides* | EU929071 | EU929074 | EU929077 |
| Asikkala virus CZ/Beskydy/412/2010/Sm | ASIV | Czech | 2010 | *Sorex minutus* | KC880341 | KC880344 | KC880347 |
| Amur virus SC-1 | ASV | Korea | 1997 | *Apodemus peninsulae* | AY675349 | AY675353 | DQ056292 |
| Bayou virus HV F0260003 | BAYV | USA | 1993 | *Homo sapiens* | GQ200820 | GQ244521 | - |
| Black Creek Canal virus | BCCV | USA | 1994 | *Sigmodon hispidus* | L39949 | L39950 | - |
| Bowé virus VN1512 | BOWV | Guinea | 2012 | *Crocidura douceti* | KC631782 | NC_034406 | KC631784 |
| Brno virus 7/2012 | BRNV | Czech | 2012 | *Nyctalus noctula* | KX845678 | KX845679 | KX845680 |
| Brno virus 11/2013 | BRNV | Czech | 2013 | *Nyctalus noctula* | - | - | KR920360 |
| Bruges virus BE/Vieux-Genappe/TE/2013/1 | BRGV | Belgium | 2013 | *Talpa europaea* | KX551960 | KX551961 | KX551962 |
| Caño Delgadito virus VHV-574 | CADV | Venezuela | 1996 | *Sigmodon alstoni* | DQ285566 | DQ284451 | - |
| Castelo dos Sonhos virus AN717313/BRA300 | CASV | Brazil | 2005 | *Oligoryzomys utiaritensis* | JX443692 | JX443702 | JX443698 |
| Cao Bang virus 3 | CBNV | Vietnam | 2006 | *Anourosorex squamipes* | EF543524 | EF543526 | EF543525 |
| Choclo virus 588 | CHOV | Panama | 2000 | *Oligoryzomys fulvescens* | KX551960 | - | KT983773 |
| Dabieshan virus NC167 | DBSV | China | 2008 | *Niviventer confucianus* | AB027523 | AB027115 | DQ989237 |
| Dobrava-Belgrade virus Ano-Poroia/Afl9/1999 | DOBV | Greece | 1999 | *Apodemus flavicollis* | AJ410615 | AJ410616 | AJ410617 |
| El Moro Canyon virus RM-97 | ELMCV | USA | 1997 | *Reithrodontomys megalotis* | U11427 | U26828 | - |
| Fugong virus FG10 | FUGV | China | 2012 | *Eothenomys eleusis* | KT899701 | NC_034466 | - |
| Fusong virus Fusong-Mf-682 | FUSV | China | 2002 | *Microtus fortis* | EU072480 | EU072488 | FJ170807 |
| Gou virus LongquanRf-11-372 | GOUV | China | 2011 | *Rattus flavipectus* | KC344252 | KC344269 | - |
| Hokkaido virus Kitahiyama128S/2008 | HOKV | Japan | 2008 | *Myodes rufocanus* | AB675463 | AB676848 | - |
| Hantaan virus JS7 | HTNV | China | 2013 | *Microtus fortis* | KP970578 | KP970566 | KP896313 |
| Hantaan virus 76-118 | HTNV | South Korea | 1976 | *Apodemus agrarius* | M14626 | Y00386 | X55901 |
| Hantaan virus AA1028 | HTNV | Russia | - | *Apodemus agrarius* | AF427318 | - | - |
| Hantaan virus AP708 | HTNV | Russia | - | *Apodemus peninsulae* | AF427322 | - | - |
| Hantaan virus CGRni1 | HTNV | China | 2004 | *Rattus nitidus* | EU363812 | EU363815 | EU837275 |
| Hantaan virus YN509 | HTNV | China | 2005 | *Niviventer confucianus* | GU329991 | GU329992 | GU329993 |
| Huangpi virus Huangpi-Pa-1 | HTNV | China | 2012 | *Pipistrellus abramus* | JX473273 | - | JX465369 |
| Imjin virus Cixi-Cl-23 | MJNV | China | 2013 | *Crocidura Iasiura* | KJ420559 | NC_034557 | NC_034564 |
| Imjin virus Cl 05-11 | MJNV | South Korea | 2004 | *Crocidura lasiura* | EF641804 | EF641798 | EF641806 |
| Isla Vista virus MC-SB-47 | ISLAV | USA | 1994 | *Microtus californicus* | U19302 | U19304 | - |
| Jabora virus Akm9635 | JABV | Brazil | 2006 | *Akodon montensis* | JN232078 | - | - |
| Jeju virus 10-11 | JJUV | South Korea | 2010 | *Crocidura shantungensis* | HQ834695 | HQ834696 | HQ834697 |
| Jemez Springs virus MSB144475 | JMSV | Canada | 2006 | *Sorex monticolus* | FJ593499 | FJ593500 | FJ593501 |
| Kenkeme virus Fuyuan-Sr-326 | KKMV | China | 2011 | *Sorex roboratus* | KJ857341 | KJ857337 | KJ857320 |
| Khabarovsk virus MF-43 | KHAV | Russia | 1989 | *Microtus fortis* | U35255 | AJ011648 | AJ011650 |
| Khabarovsk virus Fuyuan-Mm-217 | KHAV | China | 2011 | *Microtus maximowiczii* | KJ857342 | KJ857338 | KJ857321 |
| Laguna Negra virus 510B | LANV | Paraguay | 1995 | *Calomys laucha* | AF005727 | AF005728 | AF005729 |
| Laguna Negra virus LBCE/12234 | LANV | Brazil | 2010 | *Calomys callidus* | KP202359 | - | - |
| Laibin virus BT20 | LAIV | China | 2012 | *Taphozous melanopogon* | KM102247 | KM102248 | KM102249 |
| Lianghe virus Lianghe-As-217 | LHEV | China | 2010 | *Anourosorex squamipes* | JX465404 | - | JX465370 |
| Longquan virus Longquan-Ra-10 | LQUV | China | 2011 | *Rhinolophus affinis* | JX465413 | JX465398 | JX465379 |
| Longquan virus Longquan-Rm-180 | LQUV | China | 2011 | *Rhinolophus monoceros,* | JX465419 | - | JX465385 |
| Longquan virus Longquan-Rs-32 | LQUV | China | 2011 | *Rhinolophus sinicus* | JX465422 | JX465402 | JX465388 |
| Luxi virus LX309 | LXV | China | 2009 | *Eothenomys miletus* | HM756286 | HM756287 | HQ404253 |
| Magboi virus 1209 | MGBV | Sierra Leone | 2009 | *Nycteris hispida* | - | - | JN037851 |
| Makokou virus GB303 | MAKV | Gabon | 2009 | *Hipposideros ruber* | - | - | KT316176 |
| Montano virus 104/2006 | MTNV | Mexico | 2006 | *Peromyscus beatae* | AB620100 | AB620101 | AB620102 |
| Mouyassuevirus 2455 | MOYV | Ethiopia | 2014 | *Neoromicia capensis* | - | - | KX184829 |
| Mouyassuevirus KB576 | MOYV | Cote d'Ivoire | 2011 | *Neoromicia nanus* | - | - | JQ287716 |
| Mouyassuevirus KB577 | MOYV | Cote d'Ivoire | 2011 | *Neoromicia nanus* | - | - | KJ000540 |
| Necocli virus HV-O0020002 | NECV | Colombia | 2011 | *Zygodontomys brevicauda* | KF481954 | KF494345 | KF735065 |
| New York virus RI-1 | NYV | USA | 1994 | *Homo sapiens* | U09488 | U36801 | - |
| Nova virus MSB95703 | NVAV | Hungary | 1999 | *Talpa europaea* | FJ539168 | HQ840957 | FJ593498 |
| Nova virus BE/Zingem/TE/2015/2 | NVAV | Belgium | 2015 | *Talpa europaea* | KX512425 | KX512431 | KX512437 |
| Oro hantavirus TK126521 | OROV | Mexico | 2004 | *Oryzomys couesi* | EF534079 | EF534080 | - |
| Oxbow virus Ng1453 | OXBV | USA | 2003 | *Neurotrichus gibbsii* | FJ539166 | FJ539167 | FJ593497 |
| Prospect Hill virus PH1 | PHV | USA | 1984 | *Microtus pennyslvania* | Z49098 | X55129 | EF646763 |
| Puumala virus Sotkamo | PUUV | Finland | 1980 | *Myodes glareolus* | X61035 | NC_005223 | NC_005225 |
| Puumala-like virus Fusong-Cr-247 | PUUV | China | 2002 | *Clethrionomys rufocanus* | EF442087 | EF442093 | - |
| Qianhushan virus YN05-284 | QHSV | China | 2005 | *Sorex cylindricauda* | GU566023 | GU566022 | GU566021 |
| Quezon virus clone/MT1720/1657 | QZNV | Philippines | 2009 | *Rousettus amplexicaudatus* | KU950713 | KU950714 | KU950715 |
| Rio Mamore hantavirus HTN-007 | RIOMV | Peru | 1996 | *Oligoryzomys microtis* | FJ532244 | FJ608550 | FJ809772 |
| Rio Mamore hantavirus OM-556 | RIOMV | Bolivia | 1996 | *Oligoryzomys microtis* | U52136 | - | - |
| Rio Segundo hantavirus RMx-Costa-1 | RIOV | Mexico | 1989 | *Reithrodontomys mexicanus* | U18100 | - | - |
| Rockport virus MSB57412 | RKPV | USA | 1986 | *Scalopus aquaticus* | HM015218 | HM015219 | - |
| Sangassou virus Saaremaa/160V | SANGV | Estonia | 1996 | *Apodemus agrarius* | AJ009773 | AJ009774 | AJ009778 |
| Sangassou virus SA14 | SANGV | Guinea | 2004 | *Hylomyscus simus* | JQ082300 | JQ082301 | JQ082302 |
| Seoul virus HR80-39 | SEOV | South Korea | 1982 | *Rattus norvegicus* | AY273791 | S47716 | X56492 |
| Seoul virus L99 | SEOV | China | 1984 | *Rattus losea* | AF288299 | AF288298 | AF288297 |
| Seoul virus SR11 | SEOV | Japan | 1982 | *Rattus norvegicus* | M34881 | M34882 | - |
| Seoul virus SIN/06(RN41) | SEOV | Singapore | 2006 | *Rattus norvegicus* | GQ274944 | GQ274942 | - |
| Seoul virus ERIZE-ST-DIZIER/Rn | SEOV | France | 2014 | *Rattus norvegicus* | KX064274 | - | - |
| Seoul virusHumber | SEOV | United Kingdom | 2012 | *Rattus norvegicus* | KM948598 | JX879768 | JX879770 |
| Seoul virus Seoul-Baxter/NYC-D17 | SEOV | USA | 2013 | *Rattus norvegicus* | KJ950866 | KJ950862 | KJ950870 |
| Serang virus Jurong/TJK/06(RT49) | SERV | Singapore | 2006 | *Rattus tanezumi* | GQ274940 | GQ274938 | - |
| Serang virus Rt60/2000 | SERV | Indonesia | 2000 | *Rattus tanezumi* | AM998808 | AM998807 | AM998806 |
| Sin Nombre virus NM R11 | SNV | USA | 1993 | *Peromyscus maniculatus* | L37904 | L37903 | L37902 |
| Seewis virus mp70 | SWSV | Switzerland | 2006 | *Sorex araneus* | EF636024 | - | EF636026 |
| Thailand virus Thailand 741 | THAIV | Thailand | 1985 | *Bandicota indica* | AB186420 | L08756 | - |
| Thottapalayam virus VRC66412 | TPMV | India | 1971 | *Suncus murinus* | AY526097 | NC_010708 | NC_010707 |
| Thottapalayam virus Yuhuan-Sm-22 | TPMV | China | 2011 | *Suncus murinus* | KJ420565 | KJ420547 | KJ420573 |
| Topografov hantavirus Ls136V | TOPV | Russia | 1994 | *Lemmus sibiricus* | AJ011646 | AJ011647 | AJ011649 |
| Tula virus Moravia/5302Ma/94 | TULV | Czech | 1994 | *Microtus arvalis* | NC_005227 | NC_005228 | NC_005226 |
| Tula virus Sen05/205 | TULV | Germany | 2005 | *Microtus arvalis* | EU439951 | EU439962 | - |
| Xuan son virus F42682 | XSV | Vietnam | 1997 | *Hipposideros pomona* | KF704709 | KJ000538 | KF704714 |
| Xuan son virus F44580 | XSV | Vietnam | 1999 | *Hipposideros pomona* | KF704710 | - | KF704715 |
| Xuan son virus F44583 | XSV | Vietnam | 1999 | *Hipposideros pomona* | KF704711 | - | KF704716 |
| Xuan son virus F44601 | XSV | Vietnam | 1999 | *Hipposideros pomona* | KF704712 | KJ000539 | KF704717 |
| Xuan son virus VN1982B4 | XSV | Vietnam | 2012 | *Hipposideros pomona* | KC688335 | KU976427 | JX912953 |
| Yakeshi virus Yakeshi-Si-210 | YKSV | China | 2006 | *Sorex isodon* | JX465423 | JX465403 | JX465389 |
| Yuanjiang virus Yuanjiang-Mf-13 | YUJV | China | 2007 | *Microtus fortis* | FJ170795 | KJ857333 | KJ857316 |
